# Viruses under mechanical force: Implications and applications

**DOI:** 10.1016/j.virusres.2026.199767

**Published:** 2026-06-19

**Authors:** Mauricio G. Mateu

**Affiliations:** Centro de Biología Molecular Severo Ochoa (CSIC-UAM), Universidad Autónoma de Madrid, 28049 Madrid, Spain

**Keywords:** Virus, Virion, Capsid, Atomic force microscopy, Mechanical force, Stiffness, Strength, Brittleness, Material fatigue, Structure and dynamics, Viral infection

## Abstract

Viruses are evolving biological entities whose perpetuation depends on their adaptive response to many selection pressures acting on them. Viruses are also solid objects that show some degree of deformability, brittleness, and strength when subjected to mechanical forces. Atomic force microscopy (AFM) allows the controlled application of mechanical force on individual virions, capsids, and viral components under close to physiological conditions. In this way, the structure, dynamics, and mechanical properties of virus particles, and their changes by the action of biologically relevant effectors, can be investigated at the single-particle level. AFM-based studies have shown that the mechanical properties of some viruses can have an adaptive value for virus survival, that mechanical forces are among the selection pressures some viruses may be confronted with, and that some viruses can exert mechanical forces that also constitute adaptive traits. AFM studies of viruses under mechanical force are also providing new insights into the relationships between virus structure and dynamics, physical properties, and biological function, even in those cases where natural mechanical forces may not be involved. The knowledge being acquired on virus biomechanics is contributing to understand virus biology and to the development of new biomedical or nanotechnological applications. The present review is addressed to a general readership of virologists, and attempts to summarize the results of over 20 years of studies that have used AFM, in combination with other biophysical, biochemical and/or biological approaches, to explore the connections between virus biology and mechanical force.

## Highlights

• Atomic force microscopy can be used to determine the mechanical properties of viruses.
• The mechanical properties of viruses may constitute biologically adaptive traits.
• Some viruses have evolved to withstand or use mechanical forces in nature.
• Mechanical forces are used to investigate virus structure, dynamics, and function.
• Discoveries on virus biomechanics can be applied in biomedicine and nanotechnology.

## 1 Introduction

Biomechanics has been defined as the application of mechanical principles to living organisms (Innocenti, 2017). Many studies have addressed the physical and/or biological effects of mechanical forces exerted by, or acting on living organisms or some of their components. To mention but one long-standing example, research on the mechanical principles that govern muscular action was first undertaken by Aristotle, Archimedes, and Leonardo da Vinci (Innocenti, 2017), and is still going strong today in exquisite molecular detail.

From a biological standpoint, countless observations support the view that the mechanical actions exerted by living beings, and their response to mechanical forces acting on them, constitute adaptive traits shaped by natural selection. One can think, for example, of the mechanical anisotropy of a tall tree in a forest: its rather stiff trunk keeps its heavy crown upright against gravity, and allows its leaves to collect enough sunlight without being overshadowed by neighbor trees; whereas the high flexibility and low brittleness of its branches make its crown, despite its large surface area, able to withstand strong winds without breaking apart. The biological implications of mechanics are manifold, and the knowledge obtained from biomechanical studies is being extensively applied in biomedicine and engineering.

Viruses are not living organisms according to prevailing criteria to define "life", but they are evolving biological entities whose function, multiplication and perpetuation depend on their adaptive response to many selection pressures acting on them. Viruses are also solid objects and, thus, they must show some degree of deformability, brittleness, and strength against disruption when subjected to mechanical forces. Those mechanical properties may have an adaptive value for virus survival, and mechanical forces may be among the selection pressures viruses are confronted with. Moreover, viruses are dynamic entities, and virus particles may themselves exert mechanical forces, which could also constitute adaptive traits. One may, thus, formulate a number of experimentally testable questions on the connection between virus biology and mechanical force:

i) what happens if a virus is subjected to mechanical force? Does it permanently deform, recover elastically, or break apart? Can the mechanical properties of viruses be quantitatively determined?
ii) do the mechanical properties of viruses constitute biologically adaptive traits? Are viruses in nature subjected to mechanical forces? Are viruses prepared to withstand, or even use to their own advantage, the natural mechanical forces that may be acting on them? Do viruses exert mechanical forces that may contribute to their survival?
iii) can the application of mechanical forces on virus particles (or viral components) under controlled conditions in the laboratory be used to learn more on their molecular structure and dynamics, physical and physicochemical properties, and biological function?
iv) what biomedical or technological applications could be derived from fundamental studies on virus biomechanics?

Until relatively recently, those questions had not even been asked. Today, the biomechanical study of viruses is a relatively mature research area. The quantitative, experimental analysis of the mechanical properties of viruses, and of the interplay between mechanical forces and viruses, has been made possible through the development of powerful single-molecule physical techniques. These include, in particular, optical tweezers and atomic force microscopy (AFM) (Bustamante, 2008; Neumann and Nagy, 2008; Kiss et al., 2020). In addition, theoretical studies on virus mechanics contemplate virtual objects with idealized virus shapes and properties. Those virus-like objects are subjected to modeling and/or molecular dynamics simulations to provide fundamental mechanics-based insights into virus structure, properties and function (Roos et al., 2010a; Bruinsma et al., 2021; Luque and Reguera, 2024).

Uses of optical tweezers (Moffit et al., 2008; Arias-González, 2024) in biology include the quantitative analysis of the mechanical properties of nucleic acids and the mechanochemical action of biomolecular motors, including viral nanomotors (Svoboda et al., 1993; Finer et al., 1994; Bustamante et al., 2003; Moffit et al., 2008; Arias-González, 2024). In a pioneering, outstanding series of researches, the mechanochemical action of the genome-packaging motor of bacteriophage ϕ29, and the mechanical forces involved in the packaging of a stiff double-stranded (ds) DNA molecule inside a preformed virus capsid, were studied using optical tweezers (Smith et al., 2001; Chemla et al., 2005). That work was continued and extended to the dsDNA packaging motors of other phages, including λ and T4 (Chemla and Smith, 2012), and to the single-stranded (ss) RNA-packaging motor of phage ϕ6 (Hanhijarvi et al., 2017). Optical tweezers have also enabled the mechanochemical study of non-viral and viral polymerases and helicases (Herbert et al., 2008; Pyle 2008; Pitre and Te Velthuis, 2021).

Most experimental studies on virus mechanics so far have been based on the use of AFM. In a pioneering study published as early as 1997, Falvo et al. mechanically manipulated individual rod-like particles of tobacco mosaic virus (TMV) in air using the cantilever of an atomic force microscope (Falvo et al., 1997). AFM-based work on the mechanical properties of viruses started in earnest only several years later, with a trend-setting study by Ivanovska, de Pablo, Schmidt, Wuite, and their collaborators that was published in 2004 (Ivanovska et al., 2004). In their study, the current standard approach for experimentally analyzing the mechanical properties of virus particles in a physiological liquid medium was established. Prolate icosahedral particles of phage ϕ29 kept in a buffer were adsorbed in different orientations on a solid, flat substrate. The viral particles were indented using the very fine stylus, or tip, located at the end of the cantilever of an atomic force microscope. The particles and the AFM tip were kept submerged in a liquid under close to physiological conditions of pH, ionic strength and temperature. AFM measurements of the force applied, and analysis of the response of the viral particles under force, allowed their stiffness, brittleness, and strength against mechanical disruption to be quantified.

The same or similar procedures have since then been applied by a number of research groups to determine the mechanical properties of virions, capsids and other components of an increasing number of virus species. Several reviews on the mechanical properties of viruses have been published. Recent ones include those by Marchetti et al., 2016; Buzón et al., 2020; Mateu 2023; Rodríguez-Espinosa et al., 2023; de Pablo 2024; de Pablo and Mateu, 2024). Most of those reviews focused on AFM methodology; mechanical parameters as physical descriptors; physical interpretations of the mechanical properties of specific viruses; and/or comparison with theoretical models or simulations to extract physical concepts that may underlie the mechanical behavior of viruses in general. Accordingly, most of those reviews, as well as many original articles, were published in specialized biophysics books or in physics- or biophysics-oriented journals that may not be widely read by virologists involved in other research areas.

The present review is addressed mainly to a general readership of virologists who may be interested in a fully updated overview on the biological implications of virus mechanics. Rather than on purely physics-based aspects, this article emphasizes the connections between virus biology and mechanical force as seen from a molecular virologist´s perspective. More specifically, it contemplates the mechanical properties of viruses as potentially adaptive traits that may confer a biological advantage. It also emphasizes the value of effector-mediated changes in the mechanical features of virus particles as signatures for biologically relevant changes in particle structure and/or conformational dynamics. Finally, it discusses some potential applications of the fundamental knowledge already acquired on virus biomechanics.

In 2012 *Virus Research* published an article that reviewed, from a biological perspective, the first 8 years (2004–2011) of AFM-based studies on the mechanical properties of viruses (Mateu, 2012). In the 14 years that have passed since then, the field of virus biomechanics, or *viromechanics,* has truly become of age. The results obtained by many research groups using AFM in combination with other techniques have provided initial answers to fundamental biology-related questions on virus mechanics (questions (i) to (iv) stated above). In this article, those questions and answers are presented and discussed in an updated and integrated form.

Please note that theoretical studies on virus mechanics, or optical tweezers-based studies on viral nanomotors (concisely referred to and referenced above) are outside the scope of this review. AFM imaging to explore virus structure is only briefly mentioned, and the reader is referred to excellent published reviews on that subject. The original studies referenced and reviewed here include the vast majority of those that used AFM, in combination with other techniques, to investigate virus mechanical properties as adaptive biological traits or as probes to study the relationship between virus structure and biological function. However, a few results obtained using theoretical approaches, optical tweezers, and/or other biophysical, biochemical or biological analyses will be briefly mentioned in cases where they are connected with AFM studies and the conclusions of the present review.

## 2 Exploring virus structure and dynamics using AFM and high-speed (HS) AFM

The atomic force microscope (Binnig et al., 1986) allows the topographic imaging of solid objects at subnanometric spatial resolution and up to subsecond temporal resolution. It also allows the application and determination of minute mechanical forces at the nanoscale (down to tens of piconewtons). In this way, the mechanical properties of a solid object can be quantified.

### 2.1 AFM for imaging viruses

**Fig. 1:**
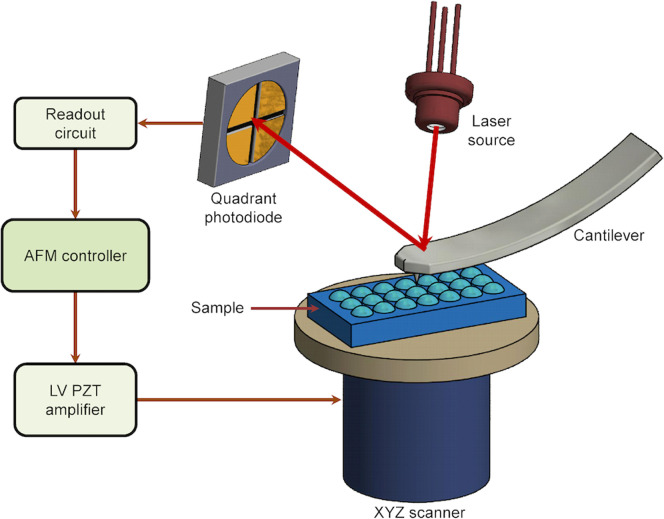
Scheme of a typical atomic force microscope. See Section 2.1 for a description of its components and function. Reproduced from Alunda and Lee, 2020, under license CC BY 4.0.

For a detailed recent review on the operation of an atomic force microscope for imaging virus particles see de Pablo, 2024. Imaging individual virions, capsids or other viral components using a typical AFM configuration (Fig. 1) involves their weak adsorption, while still submerged in a physiological buffer, onto a flat solid substrate placed on top of a piezoelectric stage. Operation of the piezoelectric stage allows the sample to be precisely displaced with subnanometer resolution along the two horizontal (*x,y*) and the vertical (*z*) dimensions. The vertical *z* distance between a fine tip-shaped probe with a radius of a few nanometers at the end of a flexible microcantilever and the sample surface is shortened until tip and sample interact weakly through an ensemble of strongly distance-dependent atomic forces. For AFM imaging of virus particles (or any other solid object) using *contact mode*, the sample is scanned along the horizontal *x* and *y* directions using computer control and a feedback loop to keep the tip-sample force constant. When the tip encounters a point of increased height, the tip-sample distance decreases, the tip-sample forces increase, and the cantilever is deflected upwards. The deflection is quantified by using a laser beam that reaches a photodiode after being reflected on the cantilever surface, and this information is then processed by the electronic system to restore the set tip-sample force and distance by changing the piezoelectric stage *z*-dimension. This *z*-displacement corresponds to the difference in height between the previous and the present sample point scanned. The height of each (*x,y*) point on the surface sample relative to the reference height (the substrate surface) is thus determined, and a topographic map of the sample contact surface is obtained.

Contact mode is not generally used for imaging mechanically soft biological objects such as virus particles that, in addition, are only weakly adsorbed to the substrate. The lateral forces exerted by the tip on the viral particle may displace, distort and/or break it. Instead, other gentler operation modes are preferred (Moreno-Herrero et al., 2002). *Jumping mode* (de Pablo et al., 1998) is frequently used for standard (not high-speed) AFM imaging. After the AFM tip contacts the sample at any (*x,y*) point, the tip is retracted, moved a very short horizontal distance to the next (*x,y*) sample point, and the tip-sample contact is re-established. In this way, lateral forces are minimized. *Tapping/non-contact* mode, in which the sample is scanned using an oscillating cantilever, is also used for both AFM and HS-AFM imaging (Section 2.2).

Determination of the structure and mechanical properties of viruses and viral components by AFM presents some weaknesses and technical limitations, but also important strengths.

AFM weaknesses as a virus structure determination technique, compared to X-ray crystallography or cryogenic electron microscopy (cryo-EM) include: i) only the topographic (surface) structure can be visualized; internal components cannot be visualized by "touching" an intact viral particle with the AFM tip. ii) AFM imaging of small organic molecules has reached atomic resolution under ultra-high vacuum using special cantilever tips and substrates (Gross et al., 2009). However, because of several technical considerations, AFM does not reach atomic resolution when virus particles or other (bio)macromolecules are imaged. iii) a suitable substrate must be found for strong enough adsorption of the virus particle without it being significantly distorted. iv) Adequate conditions, including the use of a low enough set force, should be implemented to avoid deformation of the viral particle above the resolution threshold of the technique when probed with the AFM tip.

AFM requires a careful experimental setup to circumvent a number of potential issues, either for imaging or for determination of the mechanical properties of viruses (de Pablo, 2024). An incomplete list of technical aspects that should be considered follows: i) suitable cantilevers and tips must be chosen. Larger tips limit image resolution and hamper the selective indentation of relatively small structural elements on the viral particle surface. They also result in larger dilation effects that alter the apparent horizontal dimensions of the viral particle. Sharper tips may in some cases disrupt the particle. ii) the stiffness of each cantilever used for mechanical measurements must be accurately calibrated. iii) cantilever stiffness should be carefully chosen. For example, a cantilever that is too stiff may disrupt a virus particle. A cantilever with a stiffness similar to that of the virus particle analyzed should be used to minimize error in the stiffness values determined. iv) for stiffness measurements, indentation depth should be limited to keep the viral particle under the elastic (reversible linear deformation) regime. v) indentation rate should be controlled, as it has a relevant influence on the values obtained for some mechanical parameters, such as the force required to disrupt a viral particle. vi) for biologically relevant analyses, imaging and mechanical measurements should be performed with the particles submerged in a physiological buffer; desiccation during the experiment should be prevented. vii) some virus particles may be preferentially adsorbed in a single orientation, which may limit the structural and mechanical information that can be obtained. viii) For quasi-spherical virus particles with a smooth surface, orientation of individual particles adsorbed on the substrate may be difficult to determine. ix) possible particle heterogeneity (e.g., nucleic acid-filled versus empty capsids, or intact particles versus particles missing some components) must be considered for an adequate interpretation of the results.

**Fig. 2:**
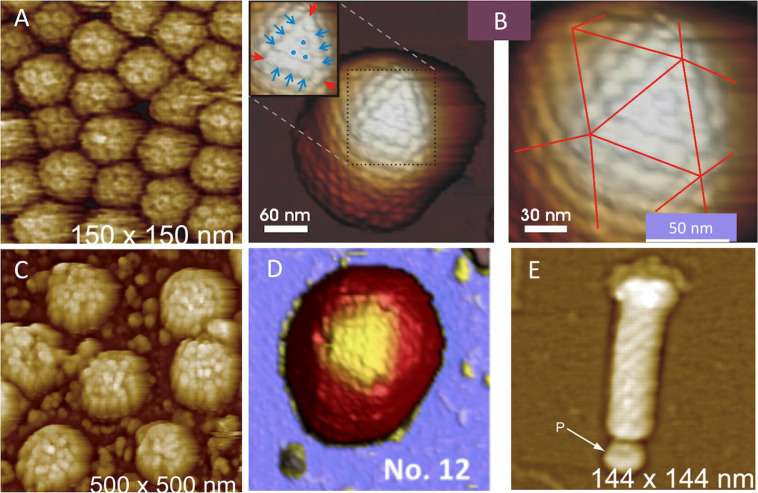
Virus particles imaged by AFM. (A) brome mosaic virus (BMV) particles (B) HSV-1 capsid. Left, image of an individual capsid; the inset depicts one facet of the icosahedral capsid; red and blue arrows point to pentons and hexons, respectively; dots mark the three central hexons. Right, close-up of the same particle. The lines delimit individual facets of the icosahedral capsid. (C) Moloney murine leukemia virions emerging from an infected cell. (D) an AdV virion missing several pentons; the concave areas at the vertices of the central triangular facet mark the original positions of three lost pentons. The capsid diameter is about 90 nm. (E) a Cyanophage tail assembly. Reproduced from Kuznetsov and McPherson, 2011, with permission by the American Society for Microbiology (ASM) (A, C, E), Roos et al., 2009, with permission by the National Academy of Sciences USA (NAS) (B), Martín-González et al., 2021, under license CC BY 4.0 (D).

AFM imaging strengths (de Pablo, 2024) include: i) the structure of virus particles and viral components can be determined under close to physiological conditions (Fig. 2): the particles can be kept submerged in a buffered liquid medium at the right pH and ionic strength, at biological temperatures, and in the presence of other biomolecules if required. ii) the structure of single particles can be determined. Thus, differences between individual particles of the same virus species can be identified. In addition, the entire distribution of individual values for a given parameter, and not only an average value, can be obtained. iii) although atomic resolution is not reached, individual capsid subunits can be visualized (Figs. 2A, 2B), and their arrangement on the virus particle can be ascertained (Fig. 2B). iv) viral particles bound to a receptor on a living cell surface or emerging from an infected cell can be visualized (Fig. 2C). v) structural changes in a same virus particle as a function of time or changing conditions can be identified (Fig. 2D)). vi) the ultrastructure of isolated complex viral components, such as phage tails, can be determined (Fig. 2E). vii) compared to other structural techniques such as X-ray crystallography, nuclear magnetic resonance spectroscopy, or cryo-EM, only a relatively small amount of diluted sample containing a few virus particles in a few microliters may be required. viii) within reason, the virus particles may not need to be extensively purified. Fine images of intact single virus particles can be obtained even in the presence of broken viral particles and/or diverse contaminants. ix) once the appropriate conditions have been found, AFM can be a relatively simple and fast imaging technique. The topographic structure of a number of individual virus particles can be determined in a matter of a few hours, even minutes.

AFM imaging using a very low set force and a gentle operation mode as summarized above is being increasingly used to determine different aspects of the architecture and molecular structure of virions, capsids, and other viral components, and virus interactions with (bio)molecules or cells (reviewed in Kuznetsov and McPherson, 2011; Marchetti et al., 2016; Ray et al., 2025) (Fig. 2). AFM provides an excellent complementary technique to X-ray crystallography, cryo-EM and other structural techniques for studying virus structure and dynamics, and their relationship with viral function.

### 2.2 HS-AFM for visualizing virus dynamics

Standard atomic force microscopes require at least several seconds to acquire a topographic image of a single virus particle. Thus, until recently AFM could be used to visualize structural changes of individual virus particles by taking successive images, but only if those changes occurred during relatively long times.

Today, the outstanding recent development of HS-AFM instruments (Ando et al., 2008; Ando, 2013; Ando, 2017) allows the visualization in real time of relatively rapid dynamic processes in which virus particles and/or their components are involved. In these instruments, the scanning speed is dramatically increased by improving the main components of an AFM microscope, including the piezoelectric stage, the microcantilever and tip, and the electronics. Depending on the sample and conditions, speeds of up to several images per second using tapping mode, even tens of images per second, have already been achieved using HS-AFM. HS-AFM shares with standard AFM the limitations and strengths mentioned in Section 2.1 above.

**Fig. 3:**
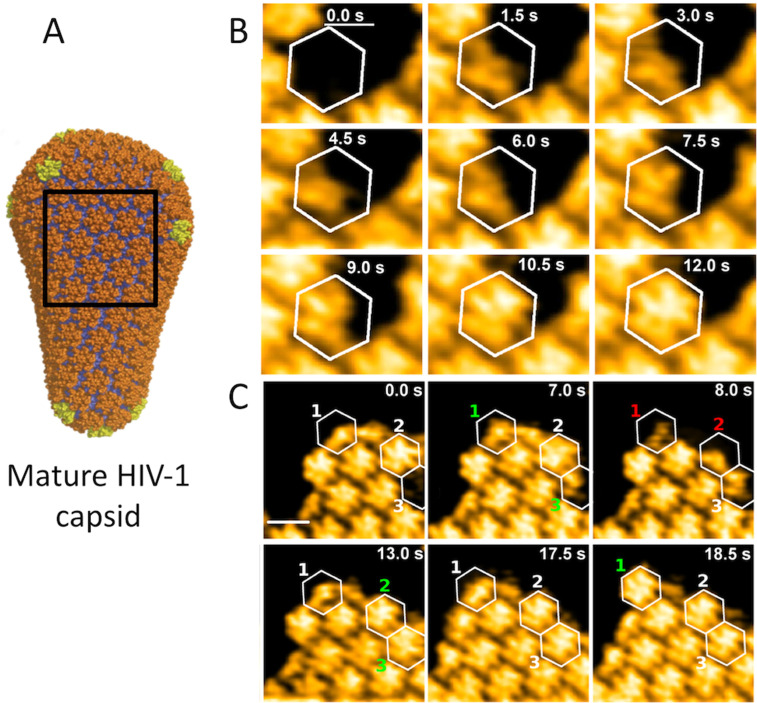
Visualization of the gradual assembly of the HIV-1 capsid lattice by HS-AFM, in real time and at single-molecule resolution. (A). Structure of the mature HIV-1 capsid. A square indicates a portion of the hexagonal lattice formed by association of capsid protein CA subunits. (B, C). Self-assembly of the mature HIV-1 capsid protein lattice visualized in real time by HS-AFM. Images are representative frames from videos of the assembly process. Time is indicated in seconds. (B) close-up view of a CA hexamer (hexagon outlined in white) being gradually assembled at the edge of a growing CA lattice patch. Scale bar (at top left image) represents 7 nm. (C) association and dissociation events during growth of a CA protein lattice patch. Scale bar (at top left image) represents 10 nm. Hexagons numbered 1, 2, 3 outline individual CA hexamers being gradually assembled through different pathways. Color coding of those numbers indicate single association events (green) or dissociation events (red). Reproduced from Valbuena et al., 2020, with permission from the American Chemical Society (ACS).

HS-AFM constitutes a unique approach for the visualization, under close to physiological conditions and in real time, of relatively fast (subsecond) structural changes and other dynamic processes in which virus particles and/or their components are involved. Virological studies using HS-AFM started only very few years ago, but they are rapidly increasing in number. Examples include the visualization in real time of: the self-assembly of the hexagonal lattice the mature human immunodeficiency virus 1 (HIV-1) capsid is made of (Valbuena et al., 2020) (Fig. 3); the self-association of the hepatitis B virus (HBV) capsid protein (Buzón et al., 2021); the budding of HIV-1 particles from infected cells (Harel et al., 2022); the conformational dynamics of influenza virus (IAV) ribonucleoprotein particles (Carlero et al., 2024); and a conformational rearrangement of the severe acute respiratory syndrome coronavirus type 2 (SARS-CoV-2) receptor-binding S protein (Saha et al., 2025).

## 3 Using AFM to determine the mechanical properties of viruses

Section 2 has briefly highlighted the usefulness of standard AFM and HS-AFM for providing insights into the molecular structure and dynamics of individual virus particles and other viral components under close to physiological conditions. Those studies are made possible by the application on single virus particles, through contact with the AFM tip, of a very weak, carefully controlled force. It naturally followed that application through the AFM tip of a considerably higher force could be used to quantify the mechanical properties of virus particles, including their stiffness, intrinsic elasticity, brittleness, strength against physical disruption, and resistance to material fatigue (Ivanovska et al., 2004; de Pablo, 2024). Table 1 includes definitions of some parameters used to quantify the mechanical properties of viruses.

**Table 1:** Virus mechanical properties terminology as used in the text.

Property	Parameter^1^	Definition
Stiffness	Spring Constant (*k_p_*)	Ratio between the force applied to a solid object and the deformation achieved. Dependent on object size and geometry
Intrinsic elasticity	Young´s Modulus of Elasticity (*E_p_*)	Ratio between the stress (pressure, force per unit area) applied and the relative deformation of a solid material. Independent of object size and geometry
Strength	Yield Force (*F_rp_*)	Maximum force a solid object withstands without being irreversibly deformed or disrupted. Dependent on loading speed
Brittleness (of a cuasi-spherical particle)	(*d_rp_/D_p_*)	For a quasi-spherical object (including most viruses), brittleness can be quantified by the ratio between the maximum deformation the object withstands *d_rp__,_*and the particle diameter *D_p_.* The lower the ratio, the higher the object´s brittleness
Material fatigue		Fatigue refers to the susceptibility of a solid object to be damaged (disrupted) when subjected to cyclic load under a force below the Yield Force. Highly dependent of the force applied, its frequency, and other experimental conditions

1 The values obtained when a virus particle is subjected to mechanical force frequently depend on the physical and chemical conditions of the experiment (e.g., pH, ionic strength, temperature, bound ions, ligand molecules, etc.). They may depend also on the instrumental conditions chosen to perform the experiments. For example, the Yield Force depends on the indentation speed; the number of load cycles required to fatigue a virus particle strongly depend on the force applied and its frequency. For virus particles, relative values obtained by comparison with those obtained for a reference viral particle under the same experimental conditions are generally more meaningful than absolute values.

### 3.1 Determination of virus stiffness and intrinsic elasticity

Many viral particles subjected to shallow enough indentations behave as ideal springs. Under such conditions, the stiffness of a virus particle can be quantified by determining its elastic constant (spring constant, *k_p_*) which, according to Hooke´s law, equals the ratio between the force applied (*F*) and the deformation achieved (*d*).

**Fig. 4:**
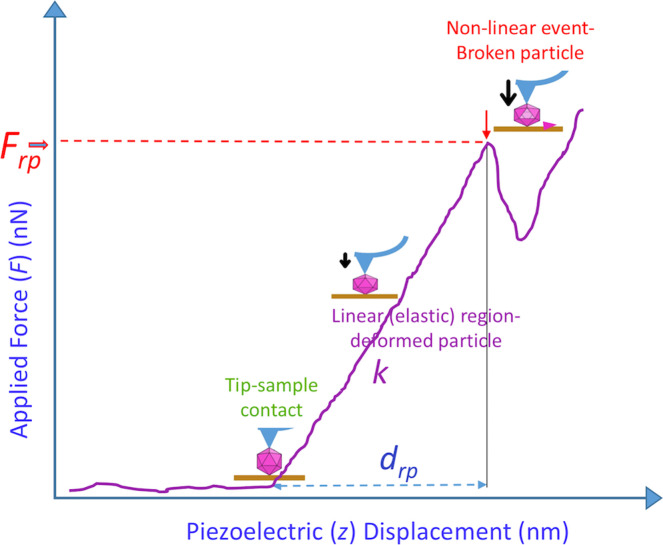
A typical force-*versus*-distance (*F-z*) curve obtained by indentation of a virus particle using an atomic force microscope to determine different mechanical properties of the viral particle. See 3.1, 3.2 for a description. Particle stiffness can be quantified by determining the spring constant of the particle *k_p_* from the slope *k* of the linear region of the *F-z* curve. This region corresponds to the elastic deformation of the viral particle. Particle strength can be quantified by determining the yield force *F_rp_*, which is the force required to trigger a non-linear event that corresponds to particle breakage (as determined by AFM imaging). Particle brittleness can be quantified by determining the distance *d_rp_,* which corresponds to the maximum deformation the particle can withstand without being broken (see text).

The viral particle is indented with a thin enough tip at the end of a microcantilever whose elastic constant (*k_c_*) had previously been determined. *F-z* curves are obtained in which the force applied (*F*) is plotted against the tip-sample vertical distance (*z*) (Fig. 4). The linear part of the *F-z* curve corresponds to the linear deformation of the system. The linear slope *k* is then determined, and the viral particle spring constant *k_p_* (along the direction of the applied force) is determined by applying Hooke´s law to cantilever and viral particle as a system of two linear springs in series (Ivanovska et al., 2004).

It must be emphasized that the elastic constant of a solid object such as a virus particle depends on both the material it is made of and its size and geometry, and it may also depend on the particle orientation relative to the vector of the applied force. The orientation of a non-spherical virus particle adsorbed on the substrate, such as the prolate icosahedral capsid of phage ϕ29, can be readily determined by AFM imaging (Ivanovska et al., 2004). The orientation of some quasi-spherical viral particles can be also determined by AFM imaging, using as reference marks the particular shapes of some conspicuous structural components. For example, prominent spikes on the 3-fold symmetry axes of the icosahedral capsids of the minute virus of mice (MVM) (Carrasco et al., 2006) and other parvoviruses; or flat protein facets on the larger icosahedral capsids of adenoviruses (AdV) (Pérez-Berná et al., 2012) (Fig. 2D), phages T7 (Hernando-Pérez et al., 2014b) and P22 (Llauró et al., 2016), and herpes simplex 1 (HSV-1) (Snijder et al., 2017) (Fig. 2B). In such cases, different *k_p_* values may be obtained depending on which region of the viral particle is located on top and directly indented. The observed anisotropic stiffness may be a consequence of particle geometry but, in some cases, it has been traced to the heterogeneity of the biological material a virus particle is made of (e.g., de Araujo Dorneles et al., 2022). Whatever the underlying cause, determination of the anisotropic stiffness of some virus particles have allowed the identification of force-related features and processes that may have biological relevance. Examples include the modulation of different conformational propensities of the MVM virion (Carrasco et al., 2008; Castellanos et al., 2012a; Section 4.6), and the existence of pre-stress in the phage ϕ29 capsid (Carrasco et al., 2011).

It must be considered also that single viral particles from a pleiotropic virus may show substantial differences in stiffness and other mechanical properties due to their structural heterogeneity. When structurally complex or pleiotropic virus particles are studied, mechanical variability among individual viral particles, and its possible biological relevance, should be considered.

To provide a quantitative value for the intrinsic elasticity of the material a viral particle is made of, irrespective of its size or geometry, another parameter can be calculated: the Young´s modulus of elasticity, *E_p_*. The *E_p_* value calculated for a viral particle or any viral component from the results obtained in indentation experiments should be interpreted with caution, as the virus biological material is not homogeneous (de Araujo Dorneles et al., 2022). However, it can be seen as an indication of the average elastic behavior of an inhomogeneous or composite biological material. As for an averaged *k_p_* value obtained for a viral particle, an averaged *E_p_* value may conceal a biologically important mechanical anisotropy (Carrasco et al., 2008; Castellanos et al., 2012a; Section 4.6). Despite the above qualifications relative, average *k_p_* and *E_p_* values have been proven especially useful to reveal global changes in stiffness and/or intrinsic elasticity of a virion or a viral capsid as a consequence of, for example, a mutation; the addition or removal of some viral component; or the interaction with a heterologous biological effector (Mateu 2012) (see 6, 7).

### 3.2 Determination of virus strength and brittleness

The stiffness of a virus particle is determined by applying a strong enough mechanical force, but not so strong as to exceed its elastic deformation limit (Section 3.1). If the applied load is further increased and the indentation of the virus particle is deep enough, a nonlinear response is frequently (though not always) detected by inspection of the *F-z* curve (Fig. 4). AFM imaging before and after the event has shown that some of those nonlinear events are the consequence of an irreversible deformation of the virus particle (Ivanovska et al., 2004; Arkhipov et al., 2009; Roos et al., 2009). Some nonlinear events arise as a result of fractures at the interfaces between viral particle subunits, leading to a plastic instead of an elastic mechanical response (Ivanovska et al., 2004; Ivanovska et al., 2011). Very frequently, nonlinear events involve the dissociation of one or a few particle subunits, eventually leading to partial or complete breakage of the viral particle (Ivanovska et al., 2004; Ivanovska et al., 2007; Castellanos et al., 2012b).

The mechanical strength of a virus particle can be quantified by measuring the force required to disrupt the physical integrity of the particle (either by irreversible deformation, fracture, loss of subunits or catastrophic failure) under specified conditions. This force, frequently termed breaking force, yield force, or rupture force, *F_rp_,* can be defined as the force at which a non-linear event occurs in the *F-z* curve obtained by deep indentation of the viral particle (Fig. 4). Special care should be exercised when comparing *F_rp_* values, as the yield force depends on the indentation speed. As some non-linear events may be the result of particle movement or tip slippage without loss of particle integrity, AFM imaging of the virus particle before and after indentation is required to make certain an irreversible deformation or disruption event has indeed occurred.

The brittleness of a virus particle can be defined as the maximum deformation it can withstand under force without being irreversibly deformed or disrupted. For a quasi-spherical virus particle of diameter *D_p_* that accepts a maximum deformation *d_rp_* (Fig. 4), the parameter *d_rp_/D_p_* can be taken as a measurement of its brittleness, with a higher value indicating a less brittle particle.

As for stiffness and intrinsic elasticity, some viral particles can be anisotropic regarding its strength and/or brittleness (Medrano et al., 2019).

### 3.3 Determination of virus resistance to material fatigue

Cyclic indentation of a single virus particle using a force well below the yield force *F_rp_* frequently results, after a high enough number of indentations, and depending on both the force and the indentation frequency, in irreversible particle structural alterations and damage. This behavior has been observed for virions or capsids of quite different virus species, such as DNA phages including ϕ29 (Ivanovska et al., 2004), λ (Ivanovska et al., 2007; Hernando-Pérez et al., 2014a), HK97 (Roos et al., 2012), and T7 (Hernando-Pérez et al., 2014b; Vörös et al., 2017); the HIV-1 capsid protein lattice (Valbuena and Mateu, 2017); rotavirus (Jiménez-Zaragoza et al., 2018); AdV (Denning et al., 2019); SARS-CoV-2 (Cardoso-Lima et al., 2021); picobirnavirus particles (Rodríguez-Espinosa et al., 2023); and the rod-like plant virus TMV (Díez-Martínez et al., 2025). These alterations may include, depending on the virus, the gradual removal of capsid subunits, eventually leading to complete particle disassembly, as in the HIV-1 capsid protein lattice; or the preferential loss of specific structural components that are weakly bound to other components, as in AdV. Those results revealed that virus particles under relatively weak but cyclic load, like many other solid objects, are subjected to material fatigue.

Quantitative evaluation of the propensity of a virus particle to disruption by material fatigue is not trivial and must take into account the force applied, the indentation frequency and other experimental conditions.

### 3.4 A comparison of the mechanical properties of virus particles

During the last two decades, the mechanical properties of virions and/or capsids of over two dozen virus species, including bacteriophages, as well as plant, animal, and human viruses have been quantitatively analyzed. Table 2 provides a (necessarily incomplete and simplified) summary of the virus species subjected to AFM-based mechanical analyses, their main structural features, the particle type(s) analyzed, some mechanical features determined, and some biological implications of their mechanical behavior. Many references are also included in this table to facilitate consultation of original studies related to each specific virus.

**Table 2:** Viruses and some of their mechanical properties determined by AFM^1^.

Virus, *family*, nucleic acid type, naked or enveloped virion	Architecture, triangulation number, diameter^2^ (nm)	Particle(s) analyzed	Elastic response^3^			Brittleness/ Breakage/ Fatigue^4^	Refs^5^
			*k* (N/m)	*E* (GPa)	Remarks		
**Plant viruses**							
TMV *Virgaviridae* ssRNA(+) naked	Helical 18 x300nm long	Virion	0.8	0.9		Relatively brittle. Tannin increases strength Susceptible to fatigue	1-3
CCMV *Bromoviridae* ssRNA(+) naked	Icosahedral T=3 28	Virion	0.20		ssRNA stiffens the article		4-7
		Capsid	0.15	0.14	At pH=5. pH- and ion-dependent stiffness	Plastic deformation at 0.3R (at pH=5). Softer but resistant to breakage at pH=6	
		Salt-stable mutant virion	0.31		Salt-stable mutant virion and capsid stiffer than wt		
		Salt-stable mutant capsid	0.19	0.19			
BMV *Bromoviridae* ssRNA(+) naked	Icosahedral T=3 28	Virion	0.21		ssRNA stiffens the particle. Macromolecular crowding modulates stiffness. ssRNA prestresses the virion	ssRNA increases strength. Macromolecular crowding modulates strength	8,9
		Capsid	0.15				
TBSV *Tombusviridae* ssRNA(+) naked	Icosahedral T=3 30	Virion	0.55				10
		Virion+Ca^2+^	0.7		Ca^2+^ increases stiffness	Ca^2+^ increases strength	
**Animal viruses**							
NV *Caliciviridae* ssRNA(+) naked	Icosahedral T=3 38	Capsid (different pHs)	0.01-0.06		pH-dependent stiffness	Relatively brittle, breakage at 0.2R; brittle at high pH	11,12
		Capsid (pH=4)	0.06	0.03			
		*Shallower indentations:* Capsid with P domain	0.3		P domain stiffens capsid. Prestressed particle	Highly resilient, plastic behavior	
		*Shallower indentations:* Capsid minus P domain	0.11	0.2			
RV *Picornaviridae* ssRNA(+) naked	Icosahedral pseudoT=3 30	Virion	0.49			Releases the RNA under force without capsid disruption; capsid very weak after RNA is released	13
		Mut. virions	0.49-0.83^6^		Mutations and antiviral drugs that fill capsid pockets stiffen the virion. Stiffening impairs infection		
		Virion+pleconaril or pirodavir	0.85				
TrV *Dicistroviridae* ssRNA(+) naked	Icosahedral pseudoT=3 30	Virion	1.46		At pH=7. pH- and Mg^2+^-dependent stiffness		14
		Capsid	0.43	0.54	At pH=7, virion stiffer than empty capsid	At pH=7, virion stronger than empty capsid	
MVM *Parvoviridae* ssDNA naked	Icosahedral T=1 25	Virion	0.6-1.4^7^		ssDNA-mediated anisotropic stiffening	Relatively brittle, but strong virion	15-20
		Capsid	0.58	1.25	Isotropic	Strong, withstands dessication	
		Mut.virions	0.5-1.3^7^		Changes in stiffness upon single amino acid substitutions modulate infectivity		
		Mut.capsids	0.5-1.3^6^	1.1-2.8		Structural determinants of mechanical strength or stiffness are different	
AAV-2 *Parvoviridae* ssDNA naked	Icosahedral T=1 25	Virion	1.6		ssDNA does not stiffen the particle	Strong, relatively brittle. ssDNA has no clear effect on strength or brittleness	21
		Capsid	1.6	0.72			
Rotavirus *Sedoreoviridae* dsRNA (segmented) naked	Icosahedral multi-layer 70	Triple layer	0.8		Stiffened by capsid outer layers	Made stronger and less brittle by capsid outer layers. Susceptible to fatigue	22
		Double layer	0.3				
		Single layer	0.2				
IBDV *Birnaviridae* dsRNA (segmented) naked	Icosahedral T=13 65	Capsid	0.35				23
		Capsid containing increasing amounts of ribonucleoprotein (RNP) complexes	0.43-0.51-0.58-0.70		dsRNA-VP3 RNP complexes stiffen the particle	RNPs modulate fatigue resistance	
hPBV *Picobirnaviridae* dsRNA (segmented) naked	Icosahedral T=1 37	Capsid	0.27				24
AdV *Adenoviridae* dsDNA naked	Icosahedral pseudoT=25 90	Virion	0.31-1.03^7^		Anisotropic stiffness. Ligand proteins modulate stiffness. Particle stiffness depends on chromatin condensation state	Chromatin decondensation pressurizes and weakens the virion. Pressurized virion susceptible to fatigue, pentons easily released, facilitates controlled genome uncoating and infection	25-34
		DNA-filled virion *vs.* empty particle	0.56 *vs.* 0.41^8^				
SV40 *Polyomaviridae* dsDNA naked	Icosahedral T=7 45	DNA-filled capsid	0.13		dsDNA does not stiffen the particle		35
		Capsid	0.12				
		Capsid+EDTA	0.09		Ca^2+^ increases stiffness	Ca^2+^ increases strength	
ZIKV *Flaviviridae* ssRNA(+) enveloped	Pleomorphic 50	Virion		0.23-0.89	*E* depends on the region indented (material heterogeneity)		36
SARS-CoV-2 *Coronaviridae* ssRNA(+) enveloped	Quasi-spherical 110	Virion	0.013		Extremely low stiffness	Extremely resistant to breakage by indentation. Susceptible to fatigue	37,38
MLV *Retroviridae* ssRNA(+) enveloped	Pleomorphic 110 Fullerene-like hexagonal mature capsid	Immature virion	0.68	0.23			39
		Mature virion	0.31	1.0	Mature virion less stiff than immature	Relatively brittle. Susceptible to fatigue	
HIV-1 *Retroviridae* ssRNA(+) enveloped	Pleomorphic 120 Fullerene-like hexagonal mature capsid, cone shaped	Immature virion	3.15	0.93			40-46
		Immature virion, Env removed	0.52				
		Env Ct removed	0.39		EnvCt responsible for higher stiffness of the immature virion		
		Mature virion	0.22	0.44	Maturation decreases virion stiffness. Decreased stiffness facilitate infection		
		Mature virion, Env removed	0.21				
		Core			Amino acid substitutions and antiviral or proviral compounds modulate stiffness	Controlled capsid disruption during endogenous genome replication facilitates uncoating. Amino acid substitutions and antiviral or proviral compounds modulate strength	
		Mature HIV-1 capsid hexagonal lattice	0.44		Amino acid substitutions and antiviral or proviral compounds modulate stiffness	Amino acid substitutions and antiviral or proviral compounds modulate strength and fatigue resistance	47-49
IAV *Orthomyxoviridae* ssRNA(-) (segmented) enveloped	Pleomorphic 100	Virion	0.04		Very low stiffness, similar to lipid envelope. Softening associated to genome release by acidification	Extremely resistant to breakage by indentation	50-53
		Envelope	0.02	0.045	Size and Temperature-dependent stiffness		
HBV *Hepadnaviridae* dsDNA enveloped	Spherical 45 Icosahedral T=4 (some T=3) capsid 30(26)	Capsid	0.09	0.37	T=4 and T=3 capsids equally stiff	Withstands large deformations. Plastic deformation at 0.6R. Susceptible to fatigue	54-56
HSV-1 *Herpesviridae* dsDNA enveloped	Pleomorphic 220 Icosahedral T=16 capsid 125	Immature capsid	0.33			Resilient; breakage at 0.36R	57-66
		Empty capsid	0.33	1.0	No effect of scaffold on *k.* Pre-stressed capsid	50% higher resilience than immature. Scaffold release and maturation increase resilience	
		DNA-filled mature capsid	0.33		No stiffening by dsDNA	dsDNA pressurizes virion. Cementing proteins strengthen the particle, which withstands pressurization. Pressurization facilitates genome uncoating and infection	
		Immature, empty or mature capsid minus pentons	0.15- 0.21		Penton-mediated stiffening	All three particles are relatively brittle. Pentons contribute to mature capsid strength	
**Bacteriophages**							
ϕ29 Tailed phage *Salasmaviridae* dsDNA naked	Prolate icosahedral T=3, Q=5 42 × 54(head) short tail	Virion				dsDNA pressurizes virion. Strong capsid withstands pressurization. DNA ejected on dessication	67-71
		Empty prohead	0.07-0.19	1.8	Anisotropic stiffness. Prestressed particle	Relatively brittle. Breakage at 0.25R. Susceptible to fatigue. Collapse on dessication	
λ Tailed phage *unassigned family* dsDNA naked	Icosahedral T=7 65(head) long tail	Virion	0.23		DNA-mediated stiffening	dsDNA pressurizes virion. Strong particle withstands pressure. Pressurization facilitates genome uncoating. Cementing proteins strengthen the particle	72-75
		DNA-free capsid	0.13	1.0	Brittle. Breakage at 0.2R. Susceptible to fatigue		
		Virion with shortened DNA	0.13			Internal pressure reduced, genome uncoating and infection impaired	
HK97 Tailed phage *Aoguangviridae* dsDNA naked	Icosahedral T=7 65(head) long tail	Prohead-I	0.018	<0.3	Stiffness varies during particle maturation	Resilient, withstands large deformations	76
		Prohead-II	0.12	0.3		Brittle, breaks at 0.2R	
		EI	0.11	0.6		Breaks at 0.3R. Less resistant to fatigue than prohead-II	
		Head-II	0.11	1.0		Covalent crosslinks strengthen the mature (Head-II) particle. Head II as brittle as EI, but more resistant to fatigue	
T7 phage Tailed phage *Autographiviridae* dsDNA naked	Icosahedral T=7 55(head) very short tail	Prohead	0.10-0.24^7^		Anisotropic stiffness	Susceptible to fatigue	77-79
		Mature capsid	0.11-0.40^7^		Anisotropic stiffness Softer at higher temperature	Susceptible to fatigue. Less brittle at higher temperature	
		DNA-filled capsid	0.73			Stepwise reversible deformation under load	
T4 phage Tailed phage *Straboviridae* dsDNA naked	Prolate icosahedral T=13, Q=21 86 × 120(head) long contractile tail	Cell attachment Fiber	0.08 radial	0.02		Breaking force value suggests 3 fibers needed for resisting thermal force when attached to host cell	80
P22 phage Tailed phage *unassigned family* dsDNA naked	Icosahedral T=7 60(head) very short tail	Capsid	0.20		Capsid softened by loss of pentons and anisotropically stiffened by Dec protein	Capsid weakened by loss of pentons and anisotropically strengthened by Dec protein	81,82
		Capsid minus penton	0.15				
		Capsid plus Dec protein	0.20-0.23				
C22 phage Tailed phage *unassigned family* dsDNA naked	Icosahedral T=7 50(head) very short tail	Virion	0.02-0.16^9^		pH, ionic strength, temperature modulate stiffness		83,84
PRD-1 Phage *Tectiviridae* dsDNA naked	Icosahedral Multi-layer T=25 65 no tail	Virion	0.57		Stiffened by capsid outer layers	Strengthened by capsid outer layers	85
		DNA-filled vesicle	0.022				

1 Due to space limitations only some results/conclusions are summarized here in highly simplified form. Please refer to the original studies cited in the last column and the text of this article.

2 Dimensions are approximate.

3 Some indicated *k* and *E* numerical values are only approximate, as they were extracted from graphic representations, or are subjected to small variations depending on conditions or viral particle region indented.

4 Strength, brittleness and susceptibility to material fatigue have been quantified for particles from a limited number of virus species under quite different conditions. Those parameters are largely dependent on certain experimental settings and may have a rather limited value from a comparative virology perspective. Thus, no numerical values for those mechanical properties have been included in this Table. Please refer to the original studies provided cited in the last column and the text of this article.

5 References: 1: Zhao et al., 2008; 2: Wang et al., 2019; 3: Díez-Martínez et al., 2025; 4: Michel et al., 2006; 5: Klug et al., 2006; 6: Wilts et al., 2010; 7: Wilts et al., 2015; 8: Hernando-Pérez et al., 2016; 9: Zeng et al., 2021; 10: Llauró et al., 2015; 11: Cuellar et al., 2010; 12: Baclayon et al., 2011; 13: Valbuena et al., 2018; 14: Snijder et al., 2013a; 15: Carrasco et al., 2006; 16: Carrasco et al., 2008; 17: Castellanos et al., 2012a; 18: Castellanos et al., 2015; 19: Carrillo et al., 2017; 20: Medrano et al., 2019; 21: Zeng et al., 2017; 22: Jiménez-Zaragoza et al., 2018; 23: Mertens et al., 2015; 24: Ortega-Esteban et al., 2020; 25: Pérez-Berná et al., 2012; 26: Snijder et al., 2013b; 27: Ortega-Esteban et al., 2013; 28: Ortega-Esteban et al., 2015a; 29: Denning et al., 2019; 30: Martín-González et al., 2021; 31: Hernando-Pérez et al., 2020; 32: Martín-González et al., 2021; 33: Pérez-Illana et al., 2021; 34: de Pablo and San Martín, 2022; 35: van Rosmalen et al., 2018; 36: de Araujo Dorneles et al., 2022; 37: Kiss et al., 2021; 38: Cardoso-Lima et al., 2021; 39: Kol et al., 2006; 40: Kol et al., 2007; 41: Pang et al., 2013; 42: Ramalho et al., 2016; 43: Rankovic et al., 2017; 44: Rankovic et al., 2021; 45: Aiken and Rousso 2021; 46: Rousso and Deshpande, 2022; 47: Valbuena et al., 2015; 48: Domínguez-Zotes et al., 2022a; 49: Escrig et al., 2025; 50: Eghiaian et al., 2009; 51: Li et al., 2011; 52: Schaap et al., 2012; 53: Li et al., 2014; 54: Uetrecht et al., 2008; 55: Arkhipov et al., 2009; 56: Roos et al., 2010b; 57: Roos et al., 2009; 58: Klug et al., 2012; 59: Bauer et al., 2013; 60: Sae-Hueng et al., 2014; 61: Bauer et al., 2015; 62: Snijder et al., 2017; 63: Brandariz-Nuñez et al., 2019; 64: Brandariz-Nuñez et al., 2020; 65: Freeman et al., 2021; 66: Evilevitch & Sae-Hueng, 2022; 67: Ivanovska et al., 2004; 68: Carrasco et al., 2009; 69: Ivanovska et al., 2011; 70: Carrasco et al., 2011; 71: Hernando-Pérez et al., 2012; 72: Evilevitch et al., 2003; 73:Ivanovska et al., 2007; 74: Evilevitch et al., 2011; 75: Hernando-Pérez et al., 2014a. 76: Roos et al., 2012; 77: Hernando-Pérez et al., 2014b; 78: Vörös et al., 2017; 79: Vörös et al., 2018; 80: Ares et al., 2014; 81: Llauró et al., 2016; 82: Kant et al., 2018; 83: Sae-Ueng et al., 2020; 84: Sae-Ueng et al., 2022; 85: Azinas et al., 2018.

6 Depends on the amino acid substitution introduced.

7 Depends on the indented region.

8 Under same conditions.

9 Depends on pH, ionic strength and temperature.

Virus particles of quite different shapes (rod-like, quasi-spherical, pleomorphic, etc.), sizes (up to a 200-fold difference in volume), architectures, type of nucleic acid genome, molecular composition, properties and biological function have been analyzed. They constitute a fair sample of the structurally and functionally different viruses found in nature. The results have revealed striking differences in mechanical behavior between different virus particles, even very similar ones (reviewed in detail in Mateu 2012, 2023).

Up to 100-fold differences in stiffness have been found between different viruses, with *k_p_* values ranging from 0.013 N/m for the enveloped virion of the coronavirus SARS-CoV-2 (Kiss et al., 2021), up to 1.4 N/m for the non-enveloped virion of the parvovirus MVM (Carrasco et al., 2006). Some of those stiffness differences between virus particles could be attributed to differences in composition, size and geometry. However, the stiffness of some protein-made capsids with similar size, shape and icosahedral architecture from different virus species also spans two orders of magnitude, from 0.01 N/m for the norovirus (NV) capsid (depending on conditions) (Cuellar et al., 2010) to 1.3 N/m for some mutant MVM capsids (Castellanos et al., 2012a).

The intrinsic elasticity of the protein material a virus capsid is made of, as defined by its calculated *E_p_* value, likewise spans two orders of magnitude, from 0.03 GPa for the NV capsid (at acidic pH) (Cuellar et al., 2010) to 1.8 GPa for phage ϕ29 prohead (Ivanovska et al., 2004) and 2.8 Gpa for a mutant MVM capsid (Castellanos et al., 2012a). Even mutant virus capsids that differ in just one amino acid per subunit showed in some cases quite large differences in both stiffness (*k_p_*) and intrinsic elasticity (*E_p_*) (Carrasco et al., 2008; Castellanos et al., 2012a).

A similar situation was observed when the mechanical strength of different virus particles was compared. Ten-fold differences in the yield force *F_rp_* were found for different virus particles, with values as high as 6–7 nN being determined for phage T7 virions (Vörös et al., 2017) and HSV-1 capsids (Liashkovich et al., 2008).

Large differences in brittleness were also observed between some virus particles. For example, the capsids of the parvoviruses MVM (Castellanos, 2012b) and adeno-associated virus-2 (AAV-2) (Zeng et al., 2017), NV (at alkaline pH) (Cuellar et al., 2010), phage ϕ29 (Ivanovska et al., 2004), and the mature phage λ capsid (Ivanovska et al., 2007) are relatively brittle and withstood deformations no larger than 10–30% of the capsid diameter (*d_rp_*/*D_p_*=0.1–0.3) without breaking apart or being irreversibly deformed. In contrast, the SARS-CoV-2 virion (Kiss et al., 2021), the NV capsid (at neutral or acidic pH) (Cuellar et al., 2010) and the cowpea chlorotic mosaic virus (CCMV) capsid (Michel et al., 2006), withstood very large reversible deformations, even wall-to-wall deformations without being irreversibly disrupted.

The number and diversity of virus species whose mechanical properties have been quantitatively determined, including many mutants of a same virus species (Mateu, 2023), is probably large enough already to enumerate a few observations that seem to apply to the ensemble of structurally and functionally quite different viruses analyzed by AFM.

i) stiffness, intrinsic elasticity, strength and/or brittleness of virions or virus capsids from different virus species can present very large differences, up to at least one or two orders of magnitude.
ii) some of those large differences can be explained in terms of large differences in overall composition, size, architecture, and/or structural organization.
iii) however, very large mechanical differences are found between some virions or viral capsids, even from a same virus species, that differ in as little as one amino acid per capsid subunit, and that show virtually indistinguishable sizes, architectures and atomic structure (except for the substituted amino acid residue).
iv) comparison of the results of quantitative studies on the mechanical properties of different virus particles are consistent with the view that some of the striking differences in stiffness, intrinsic elasticity, strength and/or brittleness may be the result of biological evolution. Different viruses could have altered their mechanical behavior (e.g., increased strength or decreased stiffness) through mutation and selection in response to different pressures for adaptation to different ecological niches (Section 4).

## 4 Mechanical properties of viruses and their role in biological adaptation

AFM-based and other experimental studies with different viruses provide strong support to the view that the mechanical properties of some particular viruses constitute biologically adaptive phenotypic traits. Some of those studies are reviewed in this section.

### 4.1 dsDNA bacteriophages and HSV-1: A virion with a high mechanical strength withstands DNA-mediated pressurization required for injection of the viral genome into the host cell or cell nucleus

A powerful combination of experimental approaches using spectrophotometry, electrophoresis, cryo-EM, optical tweezers, AFM and other analytical techniques, together with theoretical modeling, has clearly established a biological role for the mechanical properties of dsDNA viruses. These include tailed phages (ϕ29, λ, HK97, T7, etc.,), and some animal viruses like HSV- 1.

Morphogenesis of those dsDNA viruses involves as a first step the assembly of an immature, relatively weak capsid devoid of nucleic acid. During virus maturation, the resistance of the assembled capsid against disruption under a point load (its yield force *F_rp_*) and/or by material fatigue is greatly increased (Roos et al., 2009, 2012; Hernando-Pérez et al., 2014a, 2014b; Sae-Ueng et al., 2014; Llauró et al., 2016; Vörös et al., 2017; Snijder et al., 2017; Kant et al., 2018; Evilevitch and Sae-Ueng, 2022).

**Fig. 5:**
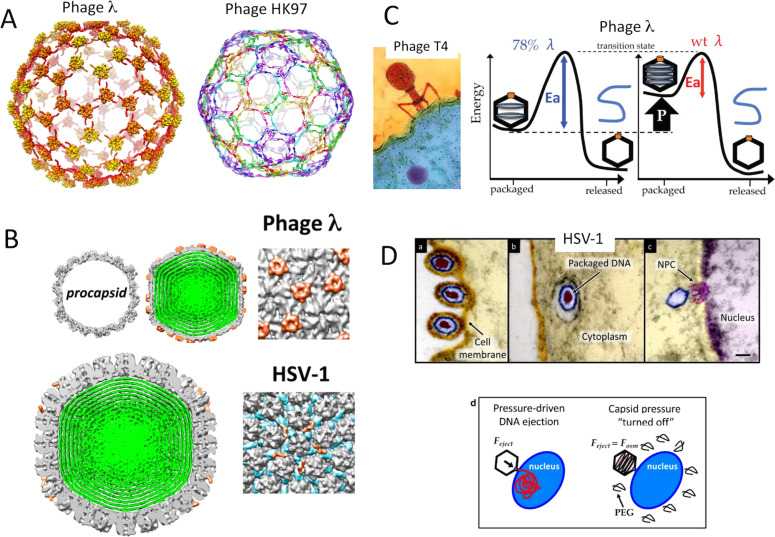
Pressurized tailed phages and HSV-1. (A) mechanical reinforcement of phage λ by bridging capsid subunits through non-covalent interactions with cementing protein gpD, and of HK97 by a chainmail of covalent bonds between capsid subunits. (B) tight packaging of the dsDNA molecule (green ribbon) inside phage λ and HSV-1 capsids. (C) DNA ejection from phages. Left, colored electron micrograph of tailed phage T4 injecting its genomic dsDNA into a bacterium. To the right from that image, simplified energy diagrams of dsDNA release from phage λ. *E_a_* is the activation free energy of the reaction. Left diagram, a virion containing a shorter dsDNA molecule is little pressurized, and *E_a_* is relatively high. Right diagram, a virion containing a full-length dsDNA is highly pressurized. The pressure *P* increases the free energy of the DNA-filled virion, but not that of the DNA-free capsid. Thus, *E_a_* decreases and DNA release is facilitated. (D) top, colored electron micrographs of HSV-1 virions adsorbed on a host cell (left image), inside the cell (center image) or bound to a nuclear pore through which the viral dsDNA has been injected into the cell nucleus (right image). Bottom left, high pressure inside the HSV-1 virion facilitates injection of its dsDNA into a cell nucleus. Bottom right, addition of polyethyleneglycol reduces the pressure difference between the virion and the external medium, impairing viral dsDNA release (see text). Reproduced from Lander et al., 2008 (A), Sae-Ueng et al., 2014 under license CC BY 4.0 (B), Bauer et al., 2015 with permission from ASM (C), and Brandariz-Nuñez et al., 2019 under license CC BY 4.0 (D).

This increase in mechanical strength is acquired through different mechanisms in different dsDNA virus species (Fig. 5A). For example, during maturation of phage HK97, a chainmail of strong covalent bonds between the capsid protein subunits is established (Huang et al., 2011). A completely different process achieves the same goal during phage λ maturation. In this case auxiliary (cementing) proteins bind the immature capsid and serve as bridges that substantially increase the number of non-covalent interactions holding the capsid subunits together (Lander et al., 2008; Hernando-Pérez et al., 2014b). HSV-1 uses other capsid-binding auxiliary proteins that strengthen mainly the weakest parts of the capsid (Klug et al., 2012; Sae-Ueng et al., 2014; Snijder et al., 2017; Evilevitch and Sae-Ueng, 2022). The fact that the capsids of different dsDNA viruses are mechanically strengthened through different mechanisms suggested that those mechanisms evolved independently in response to some common selection pressure favoring a mechanically resistant capsid.

Packaging of the dsDNA genome inside a preformed viral capsid may constitute a common selection pressure acting in different viruses for evolution of a mechanically strong mature capsid. The dsDNA molecule has a persistence length comparable to the diameter of a virus capsid. Bending the dsDNA molecule during genome packaging to fit into the viral capsid is, thus, an energetically costly process. Moreover, the viral capsid central cavity is just large enough to contain the genomic dsDNA molecule in hydrated form, which results in a very high packing density (Fig. 5B). During maturation of those viruses, a virally-encoded nanomotor uses the chemical energy provided by ATP to mechanically force the packaging of the long and stiff viral dsDNA molecule into the preformed capsid through a specific opening (Smith et al., 2001; Chemla and Smith, 2012). As a consequence, the viral particle becomes highly pressurized. The internal pressure may reach tens of atmospheres (Smith et al., 2001; Evilevitch et al., 2003; Ivanovska et al., 2007; Gelbart and Knobler 2009; Hernando-Pérez et al., 2012; Bauer et al., 2013), and exerts a strong outward mechanical force on the capsid inner wall that tends to break the capsid apart.

In principle, several evolutionary strategies could be envisaged for adaptation of the dsDNA viruses to the selection pressure imposed by the tight packaging of their stiff viral genome. The virus could have evolved a larger capsid cavity, or a streamlined, shorter viral genome. In both cases the packing density and, thus, the internal pressure would be lower, relieving the need to evolve a mechanically very strong capsid. Thus, one may wonder why for all these viruses the viral particle is kept highly pressurized, requiring the independent evolution of sophisticated strategies to mechanically reinforce the capsid.

The reason why a highly pressurized and mechanically strong virion has evolved may be related to the mechanism by which the dsDNA genome of those viruses is internalized into the host cell. The bacterial cells infected by the dsDNA phages have a thick wall that is difficult to penetrate. These phages have, thus, evolved a sophisticated entry mechanism based on the injection of their genome into the host cell, without being internalized themselves (Grayson and Molineux, 2007; van Raaij, 2024). Likewise, the dsDNA of HSV-1 must replicate within the nucleus of eukaryotic host cells. However, the HSV-1 capsid is too large to enter the nucleus through a nuclear pore. Thus, HSV-1 has evolved a mechanism to inject its dsDNA into the cell nucleus (Flatt and Greber, 2017).

Both for dsDNA phages and HSV-1, different experiments have shown that the internal pressure inside the viral particle greatly facilitates the injection of the dsDNA genome into the bacterial cell or the eukaryotic cell nucleus, respectively (Figs. 5C, 5D). For example, the internal pressure in phage λ was reduced in the laboratory by shortening the viral dsDNA. The lower pressure resulted in an increase in the activation energy required for the release of the dsDNA molecule (Ivanovska et al., 2007) (Fig. 5C). Likewise, polyethyleneglycol (PEG) was used to increase the osmotic pressure in the medium. This increased osmotic pressure counteracted the internal pressure in the HSV-1 particle and impaired viral dsDNA release (Bauer et al., 2013) (Fig. 5D).

Virus components can exert some mechanical effects also on the infected cells. For example, a recent study showed that injection of HSV-1 dsDNA into a cell nucleus increased chromatin stiffness and softened the nuclear lamina. The authors proposed that those effects may help improve the integrity of the infected cell nucleus, providing a biological advantage for virus replication (Evilevitch and Hohlbauch, 2022).

To sum up, there is strong evidence that some dsDNA viruses, including several tailed phages and HSV-1, have evolved a pressurized virion to facilitate the injection of the genome in the host bacterium or eukaryotic cell nucleus. The high internal pressure has, in turn, led to the evolution of independent mechanisms in different dsDNA viruses to increase the mechanical strength of the capsid against premature breakage under high internal pressure.

### 4.2 AdV: mechanical force exerted by increased internal pressure facilitates the gradual disassembly of the virion, and promotes viral genome uncoating

**Fig. 6:**
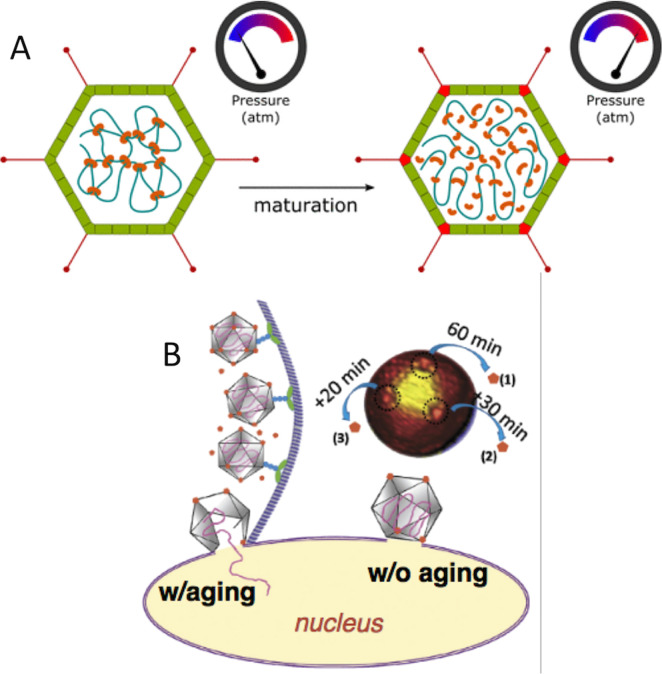
Pressure-promoted gradual disassembly of AdV. (A) left, scheme of the immature AdV virion. The chromatin is condensed, and the virion is not pressurized. Right, AdV maturation leads to chromatin decondensation. The mature virion becomes pressurized and prone to release its genomic dsDNA. (B) the mature, pressurized AdV virion is being transported to the host cell nucleus by a motor protein that walks along the cytoplasmic microtubular network. During transport, force strokes and collisions with other molecules, helped by AdV pressurization, lead to the gradual release of weakly bound pentons, allowing the controlled uncoating of the viral dsDNA and its translocation to the cell nucleus through a nuclear pore. Reproduced from Ortega-Esteban et al., 2015a with permission from ACS (A), and Martín-González et al., 2021 under license CC BY 4.0 (B).

During AdV maturation, some proteins contained inside the virion that interact with the viral dsDNA molecule are proteolytically cleaved. As a result, the viral chromatin (DNA plus DNA-binding proteins) is made less compact (Pérez-Berná et al., 2012; de Pablo and San Martín, 2022) (Fig. 6A). AFM-based indentation experiments revealed that the partially decondensed chromatin in the mature virion is less stiff than the condensed chromatin in the immature virion; and that the mature virion is stiffer than the immature virion. Positively charged polyamines can increase chromatin condensation by neutralizing the positive charges of DNA phosphates. When polyamines were added to AdV, the stiffness of the viral chromatin increased, and the stiffness of the mature virion was reduced (Ortega-Esteban et al., 2015a). Those experimental results, together with theoretical modeling, indicated that, during AdV maturation, the decondensation of chromatin results in an increase of the internal pressure in the AdV virion, up to ∼30 atm (Ortega-Esteban et al., 2013, 2015a, 2015b) (Fig. 6A). This increase in internal pressure weakens the virion: cyclic indentation with the AFM tip revealed that the mature AdV virion is more susceptible than the immature virion to the gradual loss of weakly bound penton capsid subunits, leading to partial disassembly by material fatigue (Pérez-Berná et al., 2012; Ortega-Esteban, 2013; Martín-González et al., 2019, 2021; de Pablo and San Martín, 2022) (Fig. 6B).

During infection of a host cell by AdV, the virion is transported to the cell nucleus by the action of a motor protein that "walks" through a microtubule using a brownian ratchet mechanism. This mechanism entails a discontinuous movement. Theoretical calculations suggest that force strokes during transport of the AdV particle, together with collisions with other macromolecules, generate enough mechanical stress to provoke the gradual release of pentons by material fatigue of the viral particle (Martín-González et al., 2021), as observed in indentation experiments *in vitro*. The gradual disassembly of the AdV virion facilitates the productive release of its genome into the cell nucleus through a nuclear pore (de Pablo and San Martín, 2022) (Fig. 6B).

To sum up, the results indicate that AdV has evolved a mechanically balanced mature virion. It is strong enough to preserve its integrity in the extracellular environment, but weak enough to undergo a controlled, partial disassembly process inside the infected cell. AdV maturation triggers the proteolysis-mediated decondensation of the viral chromatin, which increases the virion internal pressure, promoting the gradual release of weakly bound capsid proteins during intracellular transport of the viral particle. The partial disassembly of the viral particle, in turn, facilitates the release of the viral genome into the cell nucleus.

### 4.3 HIV-1: decreased stiffness during virion maturation facilitates virus-cell membrane fusion, and penetration of the virus core into the host cell

**Fig. 7:**
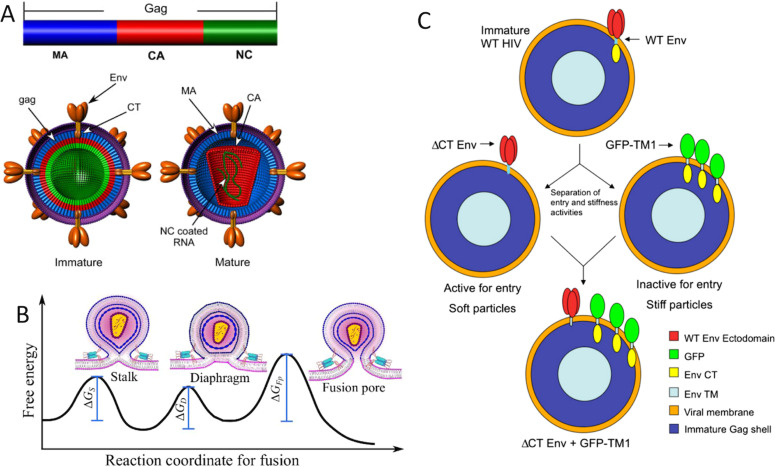
HIV-1 virion stiffness and entry into the host cell. (A) top, scheme of the immature HIV-1 capsid protein Gag, essentially made of the matrix protein (MA), the (mature) capsid protein CA, and the nucleocapsid protein (NC). Bottom, schemes of the HIV-1 immature virion (left) and of the mature virion (right). Env denotes the cell receptor-binding envelope protein. (B) the scheme depicts free energy barriers for the Env-mediated fusion of the HIV-1 lipid envelope with the cell membrane. The Env fusion subunit gp41 (represented in cyan and red) is anchored to both the viral and cell membranes. An effector-induced conformational rearrangement of gp41 exerts a mechanical pulling effect that forces the approximation of both membranes, helping to overcome the free energy barriers for their fusion. (C) Scheme of an experiment described in Section 4.3. The results showed that the Env-mediated decrease in virion stiffness that occur during its maturation is required for entry into the host cell. Reproduced from Kol et al., 2007 (A), Gorai et al., 2021 with permission from ACS (B), and Pang et al., 2013 under license CC BY 2.0 (C).

The immature HIV-1 virion is unable to enter the host cell. HIV-1 maturation involves a massive structural rearrangement (Fig. 7A) that makes the virion infectious. Infection of a host cell by a HIV-1 virion requires the fusion of the viral lipid envelope with the cell membrane, which allows the internalization of the virus core containing the RNA genome. HIV-1 virion-cell membrane fusion is promoted by the envelope (Env) protein, which is embedded in the viral membrane and specifically binds a receptor protein attached to cell membrane (reviewed by Mas and Melero, 2024) (Fig. 7B).

By using AFM, it was observed that the entry-incompetent immature HIV-1 virion is much stiffer than the entry-competent mature HIV-1 virion (Kol et al., 2007). A carefully conducted series of experiments (Pang et al., 2013) (Fig. 7C) showed that:

i) deletion of the intraparticle C-terminus (Ct) domain of the envelope protein Env both decreased the stiffness of the immature HIV-1 virion, and enabled its entry into a host cell.
ii) replacing the Env protein in the immature virion by a chimeric protein made by fusion of the green fluorescent protein (GFP) with the Env Ct (GFP-CtEnv) preserved the high stiffness of the entry-incompetent immature virion.
iii) a series of modified immature virions was then made, in which the Env Ct was deleted as in point (i) above, but that now carried different amounts of GFP-CtEnv embedded in the viral lipid envelope. The results showed that increasingly higher amounts of GFP-CtEnv led to both increasingly higher virion stiffness, and a correspondingly lower capacity to enter the host cell.

The above and other results showed that the Env Ct domain mediates the much higher stiffness of the entry-incompetent immature virion relative to the entry-competent mature virion. They also showed that a higher virion stiffness correlated with a decreased capacity for entry into the host cell.

Why does a lower stiffness facilitate entry of HIV-1 into the host cell? The answer may rest on the fact that membrane fusion is an energetically costly process (Gorai et al., 2021) (Fig. 7B). A trigger-mediated conformational rearrangement of Env results in a mechanical pulling force that brings the viral and cell membranes together, facilitating their fusion (Pang et al., 2013; Rousso and Deshpande, 2022). Thus, an increase in virion deformability (a decrease in stiffness) may allow Env-mediated viral-cell membrane fusion using a lower mechanical force.

The pulling force a fusion protein may exert, and the stiffness an enveloped virion may present, may thus constitute linked adaptive features: a virus encoding a fusion protein with a lower pulling force must possess a less stiff mature capsid to allow entry into the host cell. The pulling force exerted by Env, or any other viral fusion protein, has not been experimentally determined so far. Optical tweezers-based or AFM-based pulling experiments may allow the determination of the pulling forces exerted by viral fusion proteins. Moreover, the pulling force of a fusion protein could perhaps be modified through introduction of the appropriate amino acid substitutions using protein engineering. It could prove interesting to use the series of modified immature HIV-1 virions with increasingly higher stiffness (see point (iii) above) to investigate whether increasing or decreasing the Env pulling force would change the virion stiffness threshold required for efficient entry into the host cell.

To sum up, the results indicate that HIV-1 has evolved a virion that is made less stiff during maturation to facilitate virus-cell membrane fusion and, thus, allow the efficient delivery of the virus core into the host cell. A higher deformability of the viral particle may relieve the mechanical pulling force Env must exert to bring the viral and cell membranes close together and promote membrane fusion.

### 4.4 HIV-1: effector-mediated changes in virus core stiffness modulates entry into the cell nucleus

Once inside the cell, the HIV-1 core must enter the cell nucleus through a nuclear pore. HIV-1 mutants with stiffer capsids are impaired for nuclear entry and infection of non-dividing cells (Deshpande et al., 2024). CypA prevents both entry into the nucleus and infection by those mutants. CypA binding to mutant cores increased their stiffness, and mutations that restored nuclear entry also suppressed the CypA-mediated core stiffening (Hong et al., 2026). The results indicate that CypA may modulate nuclear entry of the HIV-1 core and viral infection by increasing core stiffness.

### 4.5 HIV-1: modulation of the mechanical strength of the viral capsid for efficient endogenous reverse transcription (ERT) of the viral genome and controlled capsid disassembly during uncoating

The RNA genome of HIV-1 is replicated by reverse transcription to DNA in the mature capsid contained within the virus core that enters in a host cell. This process is promoted by the intracellular host factor IP6 that binds the viral capsid and increases ERT efficiency. As ERT progresses, the capsid is destabilized, which facilitates viral genome uncoating (Aiken and Rousso, 2021).

AFM-based indentation on the HIV-1 core with bound IP6 during ERT showed transient changes in core stiffness. Peaks of stiffness were observed and correlated with particular stages of DNA synthesis inside the core. At a later stage, core stiffness decreased and the viral particle was disrupted, releasing the viral nucleic acid (Rankovic et al., 2017, 2021; Aiken and Rousso, 2021).

These and other results (Deshpande et al., 2025) suggested a model in which the synthesis of DNA during ERT within the confined space of the viral capsid generates transient mechanical forces that act outwards on the capsid inner wall, leading to the observed particle stiffening. Those forces would eventually result in the disruption of interactions between the capsid subunits, promoting the partial disassembly of the viral particle, and favoring viral genome uncoating. The infectivity-promoting effect of capsid-bound IP6 may be partly based on its mechanical strengthening of the viral capsid, thus restraining premature capsid disruption by the internal forces generated by the ERT process (Rankovic et al., 2021; Aiken and Rousso, 2021; Deshpande et al., 2025).

To sum up, the mechanical strength of the mature HIV-1 capsid has been tuned by being able to bind a host factor, IP6. In this way, the capsid is made strong enough to resist the internal mechanical forces exerted during ERT, but not so strong as to prevent the eventual disruption of the capsid as a result of those same forces, allowing for efficient viral genome uncoating.

### 4.6 MVM: virion anisotropic stiffness for restraining a virion-inactivating conformational transition, while allowing a transition required for virus propagation

**Fig. 8:**
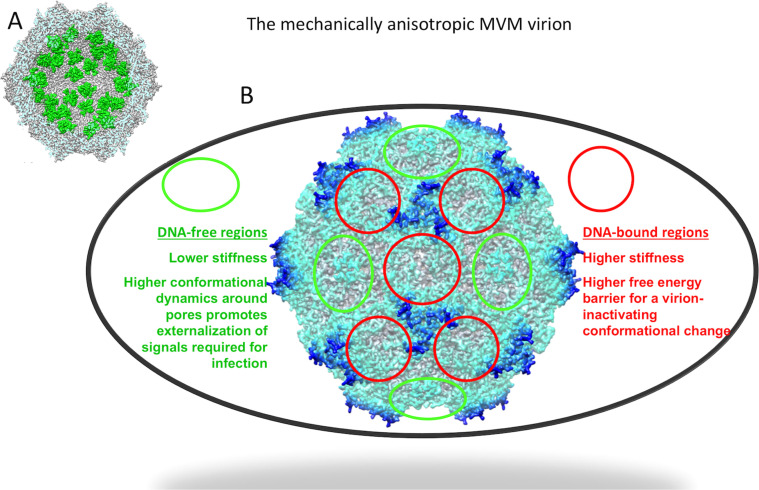
The mechanically anisotropic MVM virion and its biological relevance. (A) cross-section of the MVM structure. Structured ssDNA wedges bound to cavities at equivalent sites of the capsid inner wall are colored green. (B) Structure of the MVM virion, in which regions that show different stiffness are indicated by colored circles. Green, lower stiffness, similar to that of the ssDNA-free capsid. Red, higher stiffness as a consequence of capsid-ssDNA interactions. See Section 4.6 for a detailed description of the biological relevance of a mechanically anisotropic virion for MVM propagation.

MVM is a non-enveloped, icosahedral, single-stranded (ss) DNA virus of the parvovirus family (Fig. 8). Two different processes during the MVM infectious cycle may result in opposite selection pressures acting on this virus: i) externalization of capsid protein VP2 N-terminal (Nt) segments that carry a signal required for nuclear exit of the virion and completion of the infection process; ii) externalization of the viral ssDNA molecule, which is required for genome replication and expression in the cell nucleus, but that may occur prematurely in the extracellular medium, leading to virus inactivation (Ros et al., 2017; López-Bueno et al., 2024).

VP2 Nt externalization is, in the infected cell, a consequence of the packaging of the viral ssDNA in the parvovirus capsid by a helicase motor protein (Plevka et al., 2011; Xu et al., 2026). The limited space inside the capsid may not readily accommodate at the same time the hydrated full-length viral ssDNA and all of the VP2 Nts. Externalization of some VP2 Nts through pores located at the capsid 5-fold axes is, thus, likely promoted by a mechanical force transiently generated only while the viral ssDNA is being actively packaged to high density through one of those pores. Externalization of the VP2 Nts is associated to a subtle conformational rearrangement of the MVM capsid that, *in vitro*, can be promoted by mild heating (Reguera et al., 2004).

It is not yet clear what factor may trigger the uncoating of the MVM ssDNA genome in the infected cell. However, outside of the cell the MVM ssDNA can be prematurely released by moderate heating (Ros et al., 2006), depletion of divalent cations (Cotmore et al., 2010), particle dessication (Carrasco et al., 2009), or application of mechanical force (Strobl et al., 2023). DNA release takes place without any detectable loss of capsid integrity, which indicates it is mediated by some conformational rearrangement of the viral capsid (Castellanos et al., 2015).

It, thus, appears that MVM evolution resulted in a virion that satisfies two opposite requirements: it must allow a capsid conformational transition required for VP2 Nt externalization during morphogenesis; but it must restrain another conformational transition of the viral particle that, if unchecked, may result in the untimely release of the viral genome before it reaches the cell nucleus, leading to virus inactivation.

An AFM-based comparative analysis of the stiffness of non-mutated and mutant MVM virions and capsids supports a mechanical model on how this virus may have adapted to facilitate an infectivity-promoting conformational transition, while tempering an infectivity-impairing transition.

Indentation with the AFM tip of the nucleic acid-free capsid of wild-type (wt) MVM at or close to a 5-fold, a 3-fold, or a 2-fold axis yielded elastic constant (*k_p_*) values that were not significantly different from each other. In contrast, the ssDNA-filled virion was as stiff as the nucleic acid-free capsid when indented along a 5-fold axis, but significantly stiffer when intended along a 3-fold axis, and much stiffer when indented along a 2-fold axis. Thus, compared with the mechanically isotropic nucleic acid-free capsid, the nucleic acid-filled virion is anisotropically stiffer (Carrasco et al., 2006).

In the MVM virion, structured segments of the otherwise disordered ssDNA molecule are non-covalently bound to concavities located at equivalent sites of the capsid inner wall (Agbandje-McKenna et al., 1998) (Fig. 8A). These concavities are found closer to the capsid 2-fold axes, and far away from the pores at the capsid 5-fold axes. Deleterious amino acid substitutions that specifically removed some interactions between the capsid and the structured DNA segments invariably decreased virion stiffness to values similar to those of the DNA-free capsid, despite the full-length DNA molecule was still inside the virion (Carrasco et al., 2008). Thus, the anisotropic stiffening of the MVM virion relative to the capsid is not a result of DNA-mediated pressurization. Instead, the structured capsid-bound ssDNA segments act like internal molecular buttresses that impair the deformability of the capsid regions closer to the DNA-binding sites, i.e., the 2-fold axes and, to a lesser extent, the 3-fold axes regions. The 5-fold axes regions, being farther away from the DNA-binding sites, are not stiffened (Carrasco et al., 2008). The same mutations that decreased virion stiffness at the 2-fold and 3-fold regions by removing capsid-DNA interactions led to a decrease in the free energy barrier for the heat-induced conformational change associated to unproductive viral DNA release, and reduced viral infectivity (Fig. 8B). The activation free energy of this transition was quantitatively related with virion stiffness at the 2-fold regions, and justified using Transition State Theory (Castellanos et al., 2015).

In contrast to the effects of deleterious amino acid substitutions that removed capsid-DNA interactions, deleterious amino acid substitutions at the base of the pores at the capsid 5-fold axes invariably increased the stiffness of the pore regions, but not of the 2-fold and 3-fold axis regions located farther away (Castellanos et al., 2012a) (Fig. 8B). Those same mutations abolished the capsid conformational rearrangement associated with VP2 Nt externalization and, as a result, also abolished viral infectivity (Reguera et al., 2004). Comparison of the effects of those amino acid substitutions with other substitutions located at different capsid regions (used as controls) revealed an inextricable correlation between specific stiffening of the capsid pore regions, absence of a capsid conformational transition associated to VP2 Nt externalization, and loss of virus infectivity (Castellanos et al., 2012a).

These and other results indicate that the ssDNA-mediated anisotropic stiffening of the MVM virion has a biologically adaptive value (Fig. 8B): MVM appears to have evolved by positive selection ssDNA binding sites at specific locations of the capsid inner wall. In the virion, capsid-bound ssDNA segments act like molecular buttresses that locally rigidify the capsid regions closer to the DNA binding sites (2-fold and 3-fold regions). The DNA-mediated rigidification of those regions increases the activation free energy barrier of a conformational rearrangement, induced by heat or other means, that would lead to the premature release of the viral DNA before the virion can infect a host cell. The virus increases, thus, its chances to survive during its extracellular propagation. In turn, the capsid regions close to the 5-fold axis pores appear to have been kept free of bound DNA by negative selection, thus preserving a comparatively lower rigidity. This flexibility facilitates a conformational rearrangement of the viral particle that, during morphogenesis in the infected cell, may be triggered by the transient force exerted by the ssDNA being packaged in the viral capsid. This rearrangement results in the externalization of signals required for nuclear export of the progeny virions, and allows completion of the infectious cycle.

To sum up, the anisotropic stiffness of the MVM virion, like the anisotropic stiffness of a forest tree invoked in the Introduction, has a biologically adaptive value: viral capsid regions close to capsid-bound DNA segments are made stiff enough to restrain a conformational rearrangement that can lead to virus inactivation; whereas capsid regions farther away from bound DNA segments are kept flexible enough to facilitate a different conformational rearrangement required for virus infection (Fig. 8B).

The results summarized in this Section indicate that the mechanical properties of some structural and functionally very different viruses (including HSV-1, some dsDNA phages, AdV, HIV-1, and MVM) constitute biologically adaptive traits. The evidence available, however, does not allow to conclude that the mechanical properties of *any* virus confer a biological advantage.

## 5 Mechanical force as a selection pressure for virus survival

Viruses in nature are of course not subjected to indentation with an AFM tip. However, studies referred to in Section 4 and others indicate that some viruses can be subjected to significant mechanical stress during one or other stage of their infectious cycle. Depending on virus species and conditions, vectorial mechanical forces may act on virus particles in many different situations during their propagation between and within hosts. Some of those forces are mentioned next.

Mechanical forces in the extracellular environment: i) during transmission between organisms, virions may be subjected to mechanical forces as a consequence of osmotic shock, surface tension (passing across fluid/gas boundaries), or desiccation (Carrasco et al., 2009); ii) during fast circulation in the bloodstream or drifting in other viscous fluids, virions may be subjected to shear forces; iii) during entry into a host cell, binding of virions to multiple cell receptor molecules (Rankl et al., 2008) or, for enveloped viruses, virus-cell membrane fusion (Section 4.3) may generate mechanical pulling forces that will tend to deform the viral particle.

Mechanical forces in the intracellular environment: i) in the highly crowded intracellular medium (Rivas and Minton, 2016), virus particles are subjected to frequent collisions with other biomacromolecules; ii) during biomolecule-mediated intracellular transport, virus particles may be subjected to cyclic force strokes (Section 4.2); iii) virus particles that enter the cell nucleus (e.g., MVM or the HIV-1 core) may be squeezed during their passage through nuclear pores (Section 4.4); iv) during assembly of dsDNA viruses, forced packaging of the stiff genomic DNA molecule inside a preformed empty capsid generates an internal mechanical force that will tend to break the capsid apart (Section 4.1); v) during entry or release of the viral nucleic acid in the virion, mechanical forces may transiently arise (Section 4.6); vi) during replication or transcription of the viral genome inside the capsid of some viruses, mechanical forces acting on the capsid wall can be generated (Section 4.5); vii) both infectivity-promoting and infectivity-impairing conformational changes induced in virus particles by biological agents may result in mechanical stress. The above list of mechanical forces a virus may encounter is likely incomplete. An interesting suggestion has been made on the possibility that the mechanical properties of some viruses may have resulted from the response to the largest exposure to mechanical force those viruses may encounter during their infectious cycle.

The evidence summarized in Section 4 supports the biological role of the mechanical properties of some quite different viruses (including HSV-1, some tailed phages, AdV, HIV-1, and MVM). It also strongly suggests that the mechanical behavior of those specific viruses may have evolved in response to the selection pressure exerted by a natural mechanical force encountered or generated by the virus at some stage of its infectious cycle. The strongest case for this latter suggestion may be provided by the results of mechanical studies on dsDNA phages and HSV-1 (Section 4.1). To recapitulate, the chemical energy-driven molecular motors of those viruses exert a mechanical force that allows the packaging to high density of a long, stiff dsDNA molecule inside a viral capsid. As a result, the virions become pressurized containers, with the dsDNA molecule exerting an outwards mechanical force that facilitates injection of the viral genome into the host cell, but that also tends to break the viral particle apart. Independently evolved mechanisms, such as the establishment of a chainmail of covalent bonds or the binding of cementing proteins, make the viral capsid strong enough to resist the dsDNA-mediated mechanical force acting on it without breaking apart.

Unfortunately, experimental quantification of the mechanical forces acting on virus particles in nature is generally difficult, especially when forces in the intracellular environment may be involved. For example, it is clear that pressurization of the AdV virion promotes its gradual disassembly through material fatigue when indented with an AFM tip. Moreover, theoretical calculations do suggest that mechanical force strokes during transport of AdV may be strong and frequent enough to unbind penton subunits through fatigue of the pressurized virions (Section 4.2). However, the calculated magnitude of the force strokes inside the cell is difficult to verify by experiment. Likewise, volumetric considerations suggest that a transient mechanical force may be generated during tight ssDNA packaging in the MVM capsid (Section 4.6), but it is unclear whether the magnitude of such a force can actually promote extrusion of the VP2 Nts. Despite these qualifications, the available evidence does indicate, and further studies may fully confirm, that the observed adaptive roles of the mechanical features of quite diverse viruses are directly related to selection pressures exerted by natural mechanical forces. Again, this latter statement does not imply that the mechanical features of *any* virus have directly evolved in response to a mechanical force encountered in nature.

## 6 Genetic and effector-mediated modulation of the mechanical properties of virus particles

In addition to the studies reviewed in Section 4, many other studies have revealed changes in the mechanical properties of viral particles when these were confronted with biological effectors that modulate virus infectivity. Section 6.1 summarizes the results of studies on the structural elements and interactions that can determine the mechanical properties of virus particles, and on some genetic changes that modulate both virus infectivity and mechanical properties. Section 6.2 outline many infectivity-determining physical or chemical conditions and effector molecules that modulate the mechanical properties of virus particles. Sections 6.3 ponders cause-effect relationships between changes in virus structure, mechanical properties, and infectivity.

### 6.1 Structural determinants and genetic modulation of the mechanical properties of virus particles

A number of studies have begun to explore which specific structural elements and interactions may constitute major determinants of the mechanical features of a virion or viral capsid.

Theoretical analyses and modeling of virus mechanics have been undertaken based on elasticity theory using simplified models, finite element analysis, or molecular dynamics (MD) simulations (Roos and Wuite, 2009; Luque and Reguera, 2024). These studies are providing general, fundamental physics-based explanations for the observed mechanical behavior of virus particles, as well as insightful predictions to be experimentally tested.

In addition, AFM-based studies have experimentally addressed, at different resolution levels, the identification of specific structural determinants of the mechanical properties of virus particles, and the physical-chemical bases of their biomechanical effects. Removal of large components from a viral particle frequently resulted in conspicuous changes in stiffness, strength and/or resistance to fatigue associated to changes in viral function. In addition, comparative analyses of the mechanical properties of different intermediates of virus assembly or maturation have been undertaken using a few model viruses. Use of either approach revealed the mechanical reinforcement role played, for example, by cementing proteins in phage λ virions or covalent crosslinks in phage HK97 virions (Section 4.1); penton subunits in AdV virions (Section 4.2); the Env protein Ct domain in the immature HIV-1 virion (Section 4.3); and the N-terminus of the capsid protein that interacts with the RNA in picobirnavirusvirus-like particles (Rodríguez-Espinosa et al., 2023).

Identification of major structural determinants of the mechanical properties of viruses have been undertaken with much higher resolution through the knowledge-guided introduction of individual amino acid substitutions in capsids or virions. Over 40 different substitutions have been introduced in the MVM capsid, and their individual effects on the stiffness, intrinsic elasticity and, to a lesser extent, other mechanical properties of the viral particles have been quantified. In most cases, replacement of single amino acid residues with alanine was chosen to remove the intraparticle interactions of the original amino acid side chain, with the lowest probability to introduce other interactions or substantially alter the protein main chain conformation. Using this approach, many specific amino acid residues in the MVM capsid have been found to control the local and/or global stiffness, or the mechanical strength of the viral particle. Many of those mutation-dependent changes in mechanical behavior were linked to different mechanisms by which those mutations can impair MVM infection (Carrasco et al., 2008; Castellanos et al., 2012a, 2015; Carrillo et al., 2017; Guerra et al., 2017; Valbuena et al., 2018; Medrano et al., 2019; Luque et al., 2023). The effects of a few individual amino acid substitutions on the stiffness, strength or resistance to fatigue or other mechanical properties of viral particles from other species, including CCMV (Michel et al., 2006), human rhinovirus (RV) (Valbuena et al., 2018), AdV (van Rosmalen et al., 2017) and HIV-1 (Ramalho et al., 2016; Escrig et al., 2025), have also been quantified. In addition, the mechanical properties of three genetically different variants of the SARS-CoV-2 S protein were compared (Payam et al., 2023).

Generally speaking, the specific elements of virus particles that determine their mechanical properties are still not well defined. The results obtained so far point to a complex, context-dependent situation, but some tentative conclusions that may apply to at least some of the viruses analyzed are summarized next.

i) even the smallest structural changes in virions or capsids (e.g., the substitution of a single residue per capsid subunit) can result in large changes in the mechanical properties of the viral particle, including stiffness, intrinsic elasticity (e.g., Carrasco et al., 2008; Castellanos et al., 2012a; Carrillo et al., 2017), strength (Medrano et al., 2019; Escrig et al., 2025), and/or resistance to material fatigue (Escrig et al., 2025).
ii) changes in the mechanical properties of a virus particle are quite dependent on specific structural elements (e.g., Castellanos et al., 2012a; Carrillo et al., 2017; Medrano et al., 2019). That is, different protein molecules and different amino acid residues and the interactions they establish in the viral particle can make very different contributions to its stiffness, intrinsic elasticity, strength, or resistance to fatigue. A change in a mechanical property is not invariably achieved irrespective of which amino acid residue is replaced, which intraparticle interactions are removed or established, or where those residues and interactions are located in the viral particle.
iii) the same structural change (e.g., a same amino acid substitution) can have quite different effects on different mechanical properties of a viral particle. For example, stiffness and strength do not necessarily depend on the same viral structural elements and particle residues. Stiffness, intrinsic elasticity, brittleness and strength of a virus particle are not necessarily correlated with each other (Medrano et al., 2019).
iv) single amino acid replacements generally result in very small changes in the equilibrium (minimum free energy) conformation of a capsid or virion. However, even minute structural changes are frequently translated into substantial local and/or global changes in stiffness and/or strength of the viral particle, including changes in regions located far away from the substituted residue. Thus minor, local, specific structural changes can have a global effect on both the conformational dynamics and the mechanical behavior of the virus particle (Guerra et al., 2017; Luque et al., 2023).
v) initial experimental evidence supports two structural explanations for the global mechanical effects of minor, local changes in structure. The first explanation is that the intrinsic elasticity and/or strength of a viral capsid may depend, in part, on the number, distribution, directionality, type and energy of non-covalent interactions between atomic groups within or between subunit molecules. This hypothesis is supported, for example, by the fact that even minor structural alterations of a virion or capsid, such as a single amino acid substitution, that resulted in substantial changes in the number and/or binding energy of inter-subunit interactions had also a considerable effect on the particle intrinsic elasticity (Michel et al., 2006). A second, non-exclusive explanation, is that small changes in many atomic positions and interatomic interaction energies may be translated into very large, local or global changes in the conformational dynamics of the viral particle. This hypothesis is supported, for example, by comparative studies on linked changes in intrinsic elasticity of the MVM capsid and changes on its conformational dynamics at equilibrium as a result of individual amino acid substitutions (Luque et al., 2023; Section 7.2).

To sum up, the local and global mechanical properties of a virus particle do not necessarily correlate with each other; are very sensitive to even minor structural changes; appear to depend in complex ways on both enthalpic and entropic considerations; and are determined by specific residues and interactions in the viral particle.

### 6.2 Effector-mediated modulation of the mechanical properties of virus particles

As mentioned in Section 6.1, virus stiffness, intrinsic elasticity, strength, or resistance to material fatigue can be extremely sensitive to genetically encoded, even quite minor structural changes, such as the genetic truncation of a single amino acid side chain per capsid subunit. Likewise, a substantial number of AFM-based studies have revealed that the mechanical properties of virus particles can also be very sensitive to changes in biologically relevant physical or chemical conditions, or the binding or removal of different viral or non-viral effector molecules that modulate virus infectivity. The results of some of those studies are briefly summarized next. See Mateu, 2023 for a more detailed review and a more complete citation of original studies on this matter.

i) pH*, ionic strength, temperature*. Changes in pH, ionic strength or temperature that impaired the infectivity of some virus species had also an effect on the stiffness and/or other mechanical properties of the corresponding virus particles. For example, changes in the protonation state of CCMV (Michel et al., 2006; Wilts et al., 2010), NV (Cuellar et al., 2010), Triatoma virus (TrV) (Snijder et al., 2013a), AdV (Pérez-Illana et al., 2021), and phage C22 (Sae-Ueng et al., 2020, 2022) particles resulted in significant changes in their global stiffness. The stiffness of phage C22 also changed when the ionic strength or the temperature were increased. Heating had also an effect on both the stiffness and strength of phage T7 (Vörös et al., 2018).
ii) *macromolecular crowding*. Macromolecular crowding resulted in changes in both stiffness and strength of brome mosaic virus (BMV) particles (Zeng et al., 2021). Analysis under different conditions led to the proposal that the viral RNA exerts an internal pressure that pre-stresses the viral capsid.
iii) *osmotic pressure*. A higher osmotic pressure both impaired infectivity and increased the strength of phage λ (Evilevitch et al., 2003, 2011; Ivanovska et al., 2007) and HSV-1 (Bauer et al., 2013, 2015; Brandariz-Nuñez et al., 2019, 2020). The effect was traced to increased condensation of the viral dsDNA, which decreased the internal pressure, impaired genome ejection and, as a result, also impaired virus infectivity.
iv) *ion binding*. Mg^2+^ and/or polycations (spermine, spermidine) impaired infectivity and also decreased the stiffness of dsDNA viruses, including phages λ (Evilevitch et al., 2003, 2011; Ivanovska et al., 2007) and Φ29 (Hernando-Pérez et al., 2012), AdV (Ortega-Esteban et al., 2015a), and HSV-1 (Brandariz-Nuñez et al., 2020). This effect was, again, traced to a higher condensation of the viral nucleic acid molecule that reduced the internal pressure and impaired DNA ejection. Ca^2+^ions increased the stiffness and mechanical strength of tomato bushy stunt virus (TBSV) (Llauró et al., 2015) and simian virus 40 (SV40) (van Rosmalen et al., 2018).
v) *small molecules and host cell proteins.* Antiviral compounds pleconaril and pirodavir that bind within hydrophobic pockets in the RV capsid stiffened the virion (Valbuena et al., 2018). Tannin bound to the TMV virion both impaired virus infectivity and increased the virion strength against mechanical dissociation (Wang et al., 2019). Binding of different HIV-1 antiviral compounds to specific sites on the mature capsid resulted in changes in capsid stiffness or strength (Rankovic et al., 2018; Domínguez-Zotes et al., 2022a). Binding of the proviral IP6 factor to the HIV-1 core either stiffened or softened the particle, depending on ERT activation (Rankovic et al., 2021). Hypericin may influence the SARS-CoV-2 virion stiffness (Mariangeli et al., 2024). The differences between the mechanical properties of different norovirus virus-like particles were diminished upon fucose binding (Feng et al., 2023). These and other studies revealed an association between small-molecule binding to virus particles, small changes in structure, molecular interactions and/or conformational dynamics, changes in the mechanical properties, and modulation of virus infectivity (see Section 7).
  Binding of the HIV-1 infectivity-promoting protein CypA to the mature capsid modulated its stiffness (Liu et al., 2016; Hong et al., 2026). Regions of the AdV virion were made softer or stiffer by respectively adding integrin or defensin (Snijder et al., 2013b), and these effects were traced to the facilitation or impairment of penton release from the viral particle.
vi) *envelope-embedded or envelope-associated proteins.* The Ct domain of the Env protein in the immature HIV-1 virion led to high particle stiffness and prevented entry into the host cell; virus maturation softened the particle and enabled cell entry (Kol et al., 2007; Pang et al., 2013; Section 4.3). Removal of proteins embedded in the lipid envelope or of the matrix protein layer underneath the envelope reduced influenza A virus (IAV) stiffness (Li et al., 2011, 2014; Schaap et al., 2012; Greber 2014); these results led to a model of the structural events that occur during virus fusion to the cell membrane and ribonucleoprotein release in the cell.
vii) *capsid protein subunits and accessory viral proteins*. dsDNA-condensing viral proteins made the AdV virion less stiff. Viral cementing proteins increased both stiffness and strength of phages λ (Hernando-Pérez et al., 2014a) and P22 (Llauró et al., 2016) particles. Auxiliary proteins also stiffened the HSV-1 capsid at the vertices where the pentons are weakly bound (Sae-Ueng et al., 2014; Snijder et al., 2017; Freeman et al., 2021; Evilevitch and Sae-Ueng, 2022). Removal of pentons from the AdV virion decreased its stiffness and strength and promoted particle disassembly and genome uncoating (Pérez-Berná et al., 2012; Ortega-Esteban et al., 2013; Martín-González et al., 2021; Section 4.2).
viii) *covalent modifications of viral proteins*. Introduction of covalent bonds between capsid subunits increased the mechanical strength of the phage HK97 particle during the maturation process (Roos et al., 2012; Section 4.1). Likewise, the introduction of disulfide bonds increased the mechanical strength of the SV40 capsid (van Rosmalen et al., 2018). Chemical cross-linking by macromolecular tethers increased the stiffness of phage AP205 like-particles (Ali et al., 2024)
ix) *capsid layers*. The building of additional protein layers during morphogenesis of some viruses may contribute to strengthen the viral particle. For example, the strength and/or stiffness of rotavirus (Jiménez-Zaragoza et al., 2018) or phage PRD-1 (Azinas et al., 2018) particles increased with the number of macromolecular protein layers their capsids contained. Also, removal of the protruding (P) domain of the NV capsid decreased its strength and stiffness, which was attributed to the introduction of prestress that would stabilize the capsid.
x) *the viral nucleic acid*. For many unrelated virus species, the viral nucleic acid can modulate the stiffness, strength and/or brittleness of the virion. Examples include: ssDNA viruses (MVM, Carrasco et al., 2006, 2008); dsDNA viruses, including phages λ (Ivanovska et al., 2007), ϕ29 (Hernando-Pérez et al., 2012), T7 (Vörös et al., 2017), and PRD-1 (Azinas et al., 2018), AdV (Ortega-Esteban, 2015a), and the non-enveloped HSV-1 particle (Liaskovich et al., 2008); and ssRNA viruses, including CCMV (Michel et al., 2006), BMV (Vaughan et al., 2014), RV (Valbuena et al., 2023), and ribonucleoprotein-containing viruses such as IAV (Li et al., 2014) and infectious bursal disease virus (IBDV) (Mertens et al., 2015). The mechanism by which the virion is stiffened by the viral nucleic acid is, however, different for different nucleic acid types and virus species (compare Sections 4.1., 4.2, and 4.6). The mechanisms by which the viral ssRNA molecule inside RNA viruses may stiffen the virus particle may be related, at least for RV, to the establishment of specific interactions between sites at the capsid inner wall and structured RNA elements (Valbuena et al., 2023; Gil-Cantero et al., 2024).

### 6.3 Effector-mediated changes in mechanical properties and modulation of virus infectivity: true cause or side effect?

A vast number of studies have revealed that many effectors, including amino acid substitutions, auxiliary viral components, changes in physical or chemical conditions, and heterologous ligands can either promote or impair virus infectivity by modulating the structure and/or dynamics of virions or capsids (see, for example, Mateu, 2024). Besides, many studies, including those mentioned in Sections 4, 5, 6.1, or 6.2, have shown that many of those same biological effectors also modify the mechanical properties of those viral particles. Thus, one may ask what cause-effect relationships may exist between changes in virus structure or conformational dynamics, changes in mechanical properties, and changes in infectivity.

The results of some studies, including those reviewed in Section 4 using HSV-1, some dsDNA phages, AdV, HIV-1, or MVM, revealed that the stiffness or strength of quite different viruses constitute biologically adaptive traits that may allow them to withstand or use mechanical forces encountered in nature, or generated by the virus itself. Thus, one might be tempted to extrapolate and say that if an effector that modulates virus stiffness, strength, or fatigue resistance also modulates infectivity, it is *always* because the mechanical property in question constitutes a biologically adaptive trait; and that the effector-mediated modification of that mechanical property directly determines the survival of the virus confronted with a natural mechanical force. However, in many studies mentioned in Sections 6.1 or 6.2, and in other studies, no natural mechanical force was found associated to the observed effector-mediated changes in the mechanical properties of a virus particle. Thus, one might think instead that changes in virus particle stiffness or strength as determined by AFM *in vitro* are, in many cases, fortuitous, biologically irrelevant side effects of the effector´s true mechanism of action.

In Section 7 it is argued that an effector-mediated change in stiffness or strength of a virus particle is neither necessarily an adaptive trait associated to the action of a natural mechanical force, nor a biologically irrelevant side effect. Instead, theory and experiment together indicate that an effector-mediated change in the mechanical properties of a virus, even in those cases where it may not constitute a biologically adaptive trait in itself, provides a useful signature of some change in virus structure or conformational dynamics. A change in virus conformation or dynamics can be manifested in different ways, depending on the conceptual framework followed and the technique used for analysis: i) from the perspective of thermodynamics or kinetics, a change in structure or conformational dynamics can be reflected in changes in enthalpy, entropy, and/or activation free energy; ii) from the perspective of classic mechanics, the same change in structure or dynamics can be reflected in changes in stiffness and/or strength (see next).

## 7 Effector-mediated changes in virus particle structure or dynamics are manifested as changes in both thermodynamic or kinetic parameters, and in mechanical properties

### 7.1 Pulling and pushing on proteins and viruses

Why should effector-mediated changes in the structure or dynamics of a virus particle lead to inextricably linked changes in both stiffness and/or strength, and in thermodynamic or kinetic parameters? In previous studies on the mechanically-induced unfolding of cellular proteins or the dissociation of protein-ligand complexes through the action of a pulling mechanical force, a similar question was asked and answered (Fisher et al., 2000; Bustamante et al., 2004; Forman and Clarke, 2007; Oberhauser and Carrión-Vázquez, 2008; Rankl et al., 2008; Alsteens et al., 2009; Ferreon and Deniz, 2011; Jagannathan and Marqusee, 2013; Javadi et al., 2013).

**Fig. 9:**
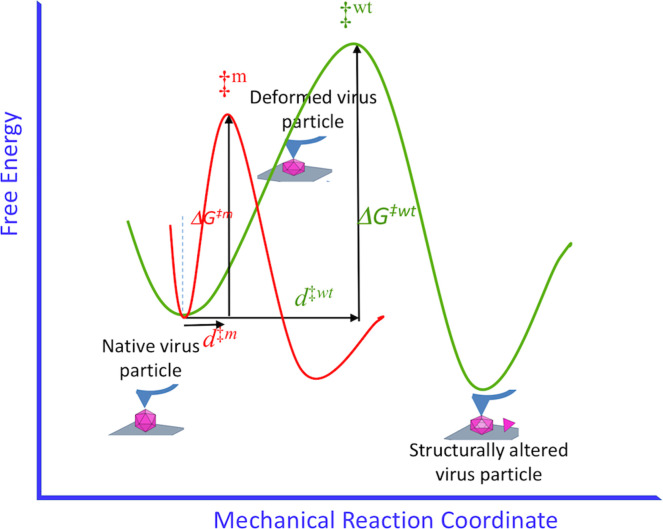
Free energy diagram of a virus particle (pink icosahedron) being deformed under a mechanical force applied by indentation with the tip of an AFM cantilever (colored blue). The particle free energy is represented as a function of the mechanical reaction coordinate between two states: a native state (a particle in its basal conformation), and a final state (e.g., a different particle conformation, a particle missing some component, or a broken particle). The green line indicates the variation of the free energy of the virus particle along the reaction coordinate in the absence of any effector (bound ligand or genetically introduced amino acid substitution) that could modify its mechanical properties. The potential energy well of the virus particle native state is represented at left. ‡*^wt^* denotes the transition state of the reaction at the free energy maximum. The potential energy well of the virus particle in the final state is represented at right. *ΔG^‡wt^* denotes the activation free energy of the reaction. *d^‡wt^* denotes the distance along the reaction coordinate between the transition state and the native state. The red line indicates the variation in free energy of the same virus particle along the reaction coordinate in the presence of an effector that modifies the particle´s mechanical properties. ‡*^m^, ΔG^‡m^, d^‡m^* respectively denote the transition state, activation free energy, and distance along the reaction coordinate between the transition state and the native state of the viral particle in the presence of that effector. In this particular example, the effector made the native state potential energy well steeper (the particle required a higher force to be deformed by a certain amount); lowered the free energy barrier of the reaction (the transition from the native state to the final state was facilitated); and shortened the distance between the transition state and the native state.

The mechanical unfolding of a protein is frequently performed by attaching one element of the protein to the substrate and another element to an AFM tip, and stretching the protein by increasing the tip-substrate separation. Likewise, the unbinding of a protein complex frequently involves attaching the protein to the substrate and the ligand to the AFM tip, approaching the tip to the substrate to allow complex formation, and then retracting the tip to disrupt the complex. When, instead of *pulling* on and stretching a protein or protein complex, one *pushes* on a virus particle with an AFM tip to determine its mechanical properties, analogous considerations may apply regarding an intimate association between: i) changes in structure and conformational dynamics, ii) changes in measured thermodynamic or kinetic parameters; and iii) changes in measured stiffness, intrinsic elasticity, brittleness, strength and/or resistance to material fatigue. A simplified model consistent with experimental results, and based on the one previously established for mechanical unfolding of proteins or unbinding of protein complexes, is outlined next. The model is applied here to a minimalist, quasi-spherical virion or capsid (such as those of MVM or RV) being deformed by a pushing force applied through an AFM tip, and assuming a two-state reaction (compare Fig. 4, Fig. 9).

Most experiments that involve pushing on a virus particle, like those that involve pulling on a protein, are kinetic experiments performed under non-equilibrium conditions. In many (but not all) of them, the viral particle is gradually deformed at a constant indentation speed, in the same way that a protein or protein complex is gradually stretched at a constant pulling speed. In both cases, the force applied increases during the process.

As a viral particle is gradually deformed with an AFM tip, atoms and atomic groups, beginning with those within the area contacted by the tip, are gradually displaced from their original positions in the equilibrium (minimum free energy) particle conformation. Because those atomic groups are bonded to other groups farther away from the initial contact area, these other groups will also be displaced from their equilibrium positions. Thus, the gradual, overall deformation of the viral particle under load will lead to an increasing number of van der Waals contacts, hydrogen bonds, ionic interactions and other electrostatic, non-covalent interactions in the native state being weakened, and eventually disrupted. As an increasing number of atomic groups are displaced, steric clashes with other groups will occur, and those clashes will have to be relieved through still more atomic displacements. In addition, the hydrophobic effect that holds clusters of hydrophobic groups together will also be weakened.

Those effects have been simulated in simplified ways in theoretical studies of virus capsid mechanics. For example, by considering that bonds between capsid elements behave as virtual springs (Vliegenhart and Gompper, 2006; Buenemann and Lenz, 2007). In the capsid equilibrium (minimum free energy) state, the springs are all relaxed. As a virtual AFM tip pushes on the capsid, the springs are more and more stretched.

A particle in which some of the non-covalent bonds that hold its components together are disrupted will be in a different conformation with an intrinsically higher free energy than its native (equilibrium) conformation. In an energy diagram (Fig. 9), the free energy of the intact particle at equilibrium is represented as a point at the bottom of the potential energy well that corresponds to the native state. The free energy of a particle in which a limited number bonds are disrupted will correspond to a point located higher on the wall of the same energy well. A mechanical force applied to the native particle will lower that free energy difference and will, thus, facilitate a change from an intact particle to a deformed particle. In the energy diagram, the force-induced change in particle conformation will be reflected also in a displacement of its "position" along the mechanical reaction coordinate (i.e., along the direction of the applied force). The final outcome will depend on whether i) the force applied does not deform the viral particle beyond its elastic limit (Section 7.2); ii) a stronger force is applied and the particle surpasses its elastic limit (Section 7.3); or iii) a relatively weak force is cyclically applied (Section 7.4).

### 7.2 Linked effector-mediated changes virus particle stiffness, and changes in conformational dynamics at equilibrium

If the indentation is shallow enough, after the force probe is withdrawn and the system is given enough time to relax, the non-covalent interactions that were stretched or disrupted will be reversibly restored; and the atomic groups in the virus particle will go back to their equilibrium positions.

From a classic mechanics perspective one can say that the elastic limit was not reached and, after the force was withdrawn, the viral particle recovered its original shape (Fig. 4). From a thermodynamics/kinetics perspective (Fig. 9), one can say that, as the particle gradually changed its conformation, the point that represents its free energy in the energy diagram was displaced along the mechanical reaction coordinate to a higher position in the native state potential energy well, but without reaching the free energy peak of the transition state (‡) of the reaction that would lead to a different thermodynamic state (e.g., an alternative particle conformation, or a broken particle). After the force is withdrawn, the particle position in the free energy landscape will return to the original (equilibrium) conformation at the bottom of the native state energy well.

One could argue that if many non-covalent interactions become gradually disrupted during a reversible deformation process, those individual bond-disruption events should be observed in the *F-z* curve as a series of multiple non-linear changes in the force value. In fact, given the very short (atomic) distances and low forces involved in the disruption of individual non-covalent interactions, in most cases those changes may be too small to be detected along the relatively noisy *F-z* curve, producing an essentially linear slope.

According to the outlined scenario, an effector-mediated change in the force required, under the conditions used, to achieve a certain particle deformation in the linear regime (a change in *k_p_*), will be related to the energy needed for a certain, reversible displacement of the atoms (away from their equilibrium positions in the particle native state). Thus, provided the particle overall size and geometry are preserved, an effector-mediated increase in stiffness (*k_p_*) may be inextricably linked to a decrease in equilibrium dynamics, reflected in a higher curvature (steepness) of the native state potential energy well (Fig. 9). Likewise, a decrease in stiffness will be linked to increased dynamics and a lower curvature (steepness) of the potential energy well.

This expectation has been qualitatively verified by experiment. Several individual substitutions of amino acids located around 5-fold axis pores in the MVM capsid impaired virus infectivity (Reguera et al., 2004). The mutant capsids were structurally nearly identical to the parent capsid, even at atomic resolution as determined by X-ray crystallography or cryo-EM (Guerra et al., 2017; Luque et al., 2023). Because the capsid size and geometry did not change upon mutation, a mutation-induced change in stiffness (*k_p_*) implies a change in intrinsic elasticity (*E_p_*). Indentation experiments using AFM showed that those mutant capsids were substantially stiffened. Accordingly, the same mutant capsids showed also diminished conformational dynamics, as determined by B-factor-based X-ray crystallography analysis or local resolution-based cryo-EM analysis, validated by hydrogen-deuterium exchange-mass spectrometry (HDX-MS) in solution (Guerra et al., 2017; Luque et al., 2023).

A linkage between changes in mechanical stiffness and changes in conformational dynamics was found also for the RV virion. AFM and other studies using RV virions with antiviral compounds bound inside capsid pockets, or carrying individual amino acid substitutions that resulted in the partial filling of those capsid pockets, revealed a correlation between reduced virus infectivity, reduced capsid pocket volume, impaired virion dynamics, and increased virion stiffness (Valbuena et al., 2018).

Likewise, betaine or certain infectivity-inhibiting compounds bound to specific sites in the mature HIV-1 capsid protein lattice led to both increased “breathing” (amplitude of the relative movements at equilibrium of capsid protein hexamers) and reduced stiffness (Valbuena et al., 2015; Domínguez-Zotes et al., 2022a).

The above results, obtained with three very different viruses, provide strong support for an inextricable relationship between changes in the equilibrium dynamics of a virus capsid, and changes in its mechanical stiffness (or more properly, intrinsic elasticity), in agreement with the model described above.

One must note, however, that such relationship may, in some cases, be blurred by the structural complexity of the virus particle and/or by certain particularities of the techniques used. As stiffness depends on size and geometry, the capsids compared should be structurally similar, as was the case in the studies mentioned above. It should also be considered that HDX-MS (or other methods in solution) provides a direction-averaged measurement of equilibrium dynamics. In contrast, stiffness is determined by AFM through indentation along the perpendicular to the viral particle surface (i.e., along the mechanical reaction coordinate, Fig. 9). If the deformability of the indented capsid region is significantly anisotropic, the *k_p_* value obtained will not provide a good estimation of stiffness along other directions. However, the relatively blunt AFM tip usually contacts a large region of the viral particle, and the deformability of such a large region may be generally expected to be fairly isotropic. Thus, the equilibrium dynamics-stiffness linkage could still be detected, as actually found for the virus models already studied.

To sum up, effector-mediated changes in the equilibrium dynamics of a virus particle (as determined by HDX-MS or other techniques) or stiffness (as determined by AFM) appear to be different manifestations of the same physical phenomenon: a structure-based change in the energy required to move atomic groups and larger structural elements in the particle a certain distance from their equilibrium positions. The detected stiffening of a virus particle in the presence of some effector may signal that, at the atomic level, such effector may be locally or globally impairing the particle equilibrium dynamics.

### 7.3 Linked effector-mediated changes in virus particle stiffness, and changes in its propensity for kinetically controlled structural rearrangements

If the particle deformation surpasses a certain threshold, reorganization and/or loss of non-covalent interactions may lead to an alternative state: a different particle conformation, a buckled (locally deformed) particle, a particle missing one or more subunits or other structural elements, or a broken particle.

From a classic mechanics perspective one can say that, as the indentation was deep enough to surpass the elastic limit, the particle was distorted or disrupted, and this outcome was reflected in a conspicuous, nonlinear change in the *F-z* curve trace (Fig. 4). From a thermodynamics/kinetics perspective, and in terms of a free energy diagram (Fig. 9) one can say that the mechanical force applied lowered the free energy barrier of the reaction enough to allow the transition from the native state to the final (distorted or disrupted) state. Or, stated in a less formal way, that the force applied was enough to overcome the free energy barrier that, in the absence of force, separated both states.

In both protein pulling and virus pushing experiments, the force required to trigger the transition between the native state and the disrupted state depends on the indentation rate, in agreement with the Bell-Evans model. This model, developed by G.I. Bell and E. Evans (Bell, 1978; Merkel et al., 1999; Evans, 2001), is closely related to the Transition State Theory (TST) by H. Eyring, M. Polanyi and M.G. Evans (Eyring, 1935). The Bell-Evans model applied to the mechanically-induced unfolding of proteins or unbinding of protein-ligand complexes states that the height of the free energy barrier between the native state and the transition state decreases in a linear fashion as the applied force increases. The unfolding or unbinding reaction rate varies exponentially with the activation energy and is, thus, force-dependent. By determining average unfolding or unbinding force values at different pulling speeds, the natural unfolding or unbinding rate at zero force, and the corresponding activation free energy and position of the transition state along the reaction coordinate, can be estimated.

An analogous situation may apply when a virus particle is subjected to a strong enough mechanical force by pushing with the AFM tip. By obtaining average yield force values at different indentation speeds, the natural rate of the particle disruption reaction at zero force, and the corresponding activation free energy and position of the transition state along the reaction coordinate, can be determined (Fig. 9). Other related approaches have been developed to extract kinetic parameters for the mechanical unfolding of proteins, and these approaches have been more recently applied to study the kinetics of transitions between different states of a viral particle in indentation experiments. For example, force-ramp experiments have been used to estimate the values of the kinetic parameters associated with the release of the genomic RNA from the RV virion (Valbuena et al., 2023).

Let us now consider a virus particle that undergoes an effector-mediated structural transition. For example, an irreversible conformational rearrangement catalyzed by a viral protease during virus maturation, or a pH-induced irreversible release of the viral nucleic acid during uncoating. Suppose that such structural transition is facilitated by removing through mutation some interactions between structural elements in that particle, and that the mutation does not change the distance between the native state and the transition state along the reaction coordinate.

If the mutant and wt particles are indented with an AFM tip, it can be expected that, in the absence in the mutant of some intra-particle interactions (all other things being equal), less mechanical force will be required to displace the atomic groups from their positions in the basal state to their positions in the transition state. In other words, the free energy barrier will be lower, and less mechanical force will be required to overcome that barrier. Thus, the yield force *F_rp_* would be expected to be lower for the mutant than for the wt particle. If the free energy difference between the transition state and the native state is lower for the mutant than for the wt particle, and the position of the transition state along the reaction coordinate is the same for both, it could be expected that the curvature of the free energy landscape from the native state to the transition state will be less pronounced in the mutant. Thus, it could be expected also that the mutant stiffness (*k_p_*) will be lower than that of the wt. Thus, under the simplified conditions stated above, an effector-mediated decrease in strength or stiffness (lower *F_rp_* and *k_p_* values) may be related to the lower free energy barrier *ΔG_p_^‡^* that, compared to the wt particle, separates the native state of the mutant particle from an alternative, conformationally altered or disrupted state. Likewise, an increase in particle strength or stiffness may reflect a higher free energy barrier.

The height of the free energy barrier of a mechanically-induced transition frequently depends on the direction of the applied force. One example is provided by the structural elements termed mechanical clamps found in shock-absorbing proteins (Carrión-Vázquez et al., 2003). Thus, the activation energy of the unfolding reaction (and, thus, the kinetic stability) of a protein determined by mechanical unfolding can frequently depend on the pulling direction, and may differ from the activation energy (and kinetic stability) determined by using a non-directional protein denaturant (e.g., heat or urea). However, as already mentioned, during indentation of a viral particle the AFM tip contacts a relatively large surface area, and a significantly anisotropic energy barrier for a transition between particle states may not be necessarily expected (Mateu 2023). For example, in a study on MVM referred to in Section 4.6, the stiffness (*k_p_* value) was determined for individual wt and mutant virions carrying single amino acid substitutions, including some that eliminated a number of capsid-viral ssDNA interactions. Those same virions were then subjected to moderate heat to induce a virus-inactivating conformational transition associated to DNA release through a capsid pore, and the rate constant for virus inactivation, *k_off_*, was determined by measuring the residual infectivity as a function of time in bulk experiments. Interestingly, a quantitative, linear correlation was found between virion stiffness *k_p_* at the 2-fold axes regions (close to where the ssDNA "wedges" are bound) and the activation free energy *ΔG_p_^‡^* of the virus inactivation reaction determined by moderate heating of MVM virions in bulk experiments (Castellanos et al., 2015).

Likewise, in a different study using many MVM mutant capsids, an inextricable association was found between increased stiffness of the capsid regions centered on the 5-fold symmetry axis pores, and decreased propensity for a biologically relevant transition between two capsid conformations, that, *in vitro*, can be induced through mild heating*,* and that is associated to externalization of VP2 Nt segments through those pores (Castellanos et al., 2012a; see Section 4.6).

It should be also noted here that some crucial aspects of virus biology involve the action of directional mechanical forces *in vivo*. Examples include virus binding to multiple receptors, virus-cell membrane fusion events, pressurized virions in which an internal mechanical force pushes radially outwards on the capsid inner wall, etc. (see Section 4). In those cases, quantitative studies of virus stability or uncoating, in which the associated energy barriers are overcome by application of a directional mechanical force, may provide biologically relevant insights that could not be obtained in experiments using thermal or chemical energy, which cannot be directionally applied.

### 7.4 Material fatigue of virus particles from a thermodynamic/kinetic perspective

The mechanism involved in the mechanical fatigue of virus particles or other protein-based complexes is still unclear. The results obtained to date are, however, consistent with a tentative simplified mechanism (Mateu 2023), outlined next.

A first indentation of a virus particle using a weak force well below the yield force *F_rp_* will disrupt only a few, weaker non-covalent bonds, including some of those that hold the capsid subunits together. If the AFM tip is retracted and enough time is allowed, all those disrupted bonds will be restored in a reversible process. If, however, a second indentation is performed using the same weak force, but before all of the bonds disrupted in the previous indentation had time to re-form, the total number of broken inter-subunit bonds will increase (Vörös et al., 2017). After a high enough number of indentations at a high enough rate, the number of inter-subunit bonds that had no time to re-form will be enough to result in the loss of individual subunits, and/or the falling apart of the viral particle.

From a classical mechanics and material science perspective one can say that the cyclic application of a mechanical force well below the yield force *F_rp_*, but at a high enough frequency, will result in material fatigue through the gradual opening and merging of "cracks" in the material. In the simplified thermodynamic/kinetic perspective adopted here, material fatigue could be seen as the result of a kinetic ratchet. In each indentation step, the force applied will lead to a particle conformation with additional broken bonds that will, thus, accumulate with the number of indentations. The cyclic application of even a small force on a virus particle may eventually overcome the free energy barrier of a reaction in which the particle will be disrupted through a gradual or catastrophic loss of molecular components.

### 7.5 Probing effector-mediated changes in the mechanical properties of virus particles to explore changes in their structure and/or dynamics

Sections 7.1 to 7.4 have reviewed the simplified conceptual basis and some experimental evidence for the intimate linkage found between changes in structure and/or dynamics, changes in thermodynamic and/or kinetic parameters, and changes in stiffness and/or strength of a virus particle. This linkage supports the value of AFM-based analysis of the mechanical properties of virus particles to explore, at the single particle level, biologically relevant, effector-mediated changes in structure and/or dynamics of virus particles.

Section 6 described quite diverse effector-mediated changes in mechanical properties of virus particles associated to virus infectivity. In many cases, the observed change in mechanical behavior was not necessarily associated to a selection pressure exerted by a natural mechanical force. It must be stressed that, in many such studies, application of mechanical force on a virus particle *in vitro* through indentation with an AFM tip was not intended to mimic any mechanical force that could be acting on that particle *in vivo*. Mechanical force was used in those studies much like chemical agents or heat are used in other studies, i.e., as a suitable *in vitro* effector to influence the thermodynamics or kinetics of the virus particle, and learn about some aspect related to its structure, dynamics and/or biological function.

In bulk experiments to study virus structure, physical properties and biological function, averaged results over a huge number of virus particles are obtained; any difference between individual virus particles, including those that could be biologically relevant, cannot be detected. Thus, the potential of AFM to study linked changes in structure/dynamics, changes in thermodynamics/kinetics, and changes in mechanical properties of *single* virus particles, one at a time, cannot be overemphasized. A few examples related to virus-cell receptor recognition or viral genome uncoating will be briefly mentioned next to illustrate this point.

In a study with RV, the attachment of single virions to multiple cell receptors was investigated by AFM (Rankl et al., 2008). In a study with reovirus particles bound to cell receptor molecules, AFM and MD simulations were used to provide quantitative insights into virus-receptor interactions at the single-particle level (dos Santos Natividade et al., 2023). In a study on AdV, a series of position-dependent events involving the gradual loss of specific pentons were detected during the partial disassembly of the individual virions induced by material fatigue (Martín-González et al., 2021; see Section 4.2). In another study on RV, significant differences between individual virions were observed regarding the partial or complete release of the genomic ssRNA molecule from different individual virions (Valbuena et al., 2023). The above-mentioned and other insights on virus biology could not have been obtained by using bulk approaches that yield results averaged over a high number of virus particles.

## 8 Some potential applications of fundamental studies on virus biomechanics

The study of virus mechanics started in earnest only some 20 years ago. However, several biomedical or nanotechnological applications of the fundamental knowledge acquired on the relationships between virus structure-function and mechanical forces have already been proposed based on experimental evidence. In this Section, a few of those practical proposals are briefly reviewed.

### 8.1 Virus mechanics and the development of antiviral drugs

Some fundamental studies on the mechanical properties of viruses, reviewed in 4.6, 7.2, have shown that substitution of specific residues in the capsids of different viruses such as MVM or human RV, and/or binding of certain organic compounds to those capsids, increased the mechanical stiffness and reduced the conformational dynamics of the viral particles, which resulted in impaired viral infection. Studies on human RV have revealed a quantitative relationship between increased virion stiffness and impaired virus infectivity (Valbuena et al., 2018). These findings open the way to develop antiviral drugs that inhibit viral infection by mechanically stiffening the viral particles, thus restraining their biologically relevant conformational dynamism.

Other fundamental studies, reviewed in 4.1, 4.2, have shown that dsDNA mediated pressurization of tailed phages and human viruses such as HSV-1 and AdV generates an internal mechanical force that promotes the ejection of the viral genome and facilitates infection. In those studies, it was found that small polycationic molecules that can neutralize the negatively charged DNA phosphates reduced the virion internal pressure, and impaired virus infectivity without having a cytotoxic effect. Those results have led to a proposal for developing antiviral drugs that inhibit infection by dsDNA viruses by decreasing the virion internal pressure (Brandariz-Nuñez et al., 2020). Such drugs could form the basis of a novel, extremely powerful antiviral approach. As the biological effect of virion pressurization depends on dsDNA length, but is independent of the nucleotide sequence of the viral DNA and the amino acid sequence of the viral genome-encoded proteins, no drug-escape mutations could possibly be selected for in the viral populations in the presence of the drug. Thus, drug-resistant variant viruses, a major problem with current antiviral drugs, could not arise during clinical use of the proposed virus pressure-reducing antiviral drugs.

### 8.2 Virus mechanics and the development of nanomaterials and nanodevices

**Fig. 10:**
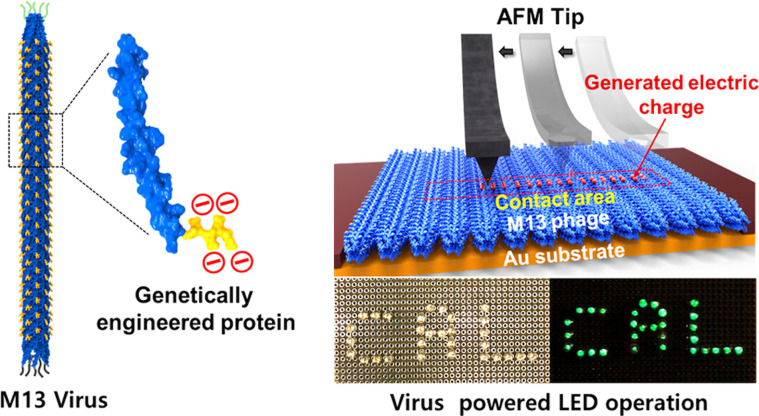
Electricity generation by a 2D array of M13 virus particles. Left, M13 phage structure, in which additional negatively charged amino acid residues were substituted by genetic engineering. (B) a 2D array of M13 phages on a gold substrate. Sliding a metal-coated AFM tip in contact with the virus array generated enough electricity to activate a LED panel (Kim et al., 2021). A similar result was obtained by the cyclic application of mechanical force on the virus array (Lee et al., 2012). Reproduced from Kim et al. (2021) under license CC BY-NC—ND 4.0.

Triboelectricity and piezoelectricity respectively refer to the generation of electricity by establishing contact with, or exerting a mechanical force on, a suitable material. In a series of remarkable studies, a virus was used to generate electrical energy. Phage M13 films were formed on a gold substrate, and a metal-coated AFM tip was used to either establish contact with the phage film (Kim et al., 2021) or apply a cyclic mechanical force on it (Lee et al., 2012). In both cases, an electrical potential was generated (Fig. 10). Moreover, the triboelectric and piezoelectric responses were increased by changing, through genetic engineering, the number of negatively charged groups on the virus particle surface. A film made of engineered M13 viruses was used to build triboelectric and piezoelectric generators that supplied enough electricity to power a liquid crystal display. This study provided proof of principle for the use of virus particles to build nanodevices that harvest mechanical energy to generate electrical energy.

In addition to electricity generation, many other applications of virus particles are being considered. For example, independent virus particles or nanostructured arrays of viral particles or capsid proteins are being explored for biomedical uses including diagnosis, vaccines, gene therapy, drug delivery, phage therapy, oncolytic virotherapy, or cell tissue regeneration. Virus particles and particle arrays are also being considered for nanotechnological uses including biosensors, confined enzyme reactors, fabrication of metal or bio-inorganic nanoparticles, antifouling coatings, nanofilters, and energy harvesting devices (as mentioned above) (recently reviewed in Mateu and Valbuena 2024). However, mechanical studies have repeatedly shown that natural virus particles and protein-derived materials are generally soft, and prone to disruption by relatively weak mechanical forces and material fatigue. Fatigue is, indeed, the main cause of failure of many materials. Thus, virus-derived particles and protein arrays could be dramatically improved as components of many useful nanomaterials or nanodevices if their mechanical strength and resistance to fatigue could be increased. Different studies reviewed in Section 6 have demonstrated the possibility to modulate the stiffness, strength and fatigue resistance of virus particles through the binding of different effector molecules, or by chemical or genetic modification. Genetic modification using protein engineering is a particularly desirable approach, as the mechanical properties of the nanomaterial would be intrinsically and permanently modified, facilitating quality control of different batches of the material, and allowing durable and fully reproducible effects.

An example of the above-mentioned possibilities is provided by applied research on nanostructured bidimensional (2D) materials, which are being explored for multiple applications. In some of those applications the material will be repeatedly subjected to mechanical forces and fatigue. For example, when used as a biocompatible coating for cell tissue growth or regeneration, an antifouling coating, or a nanofilter (Mateu and Valbuena, 2024).

**Fig. 11:**
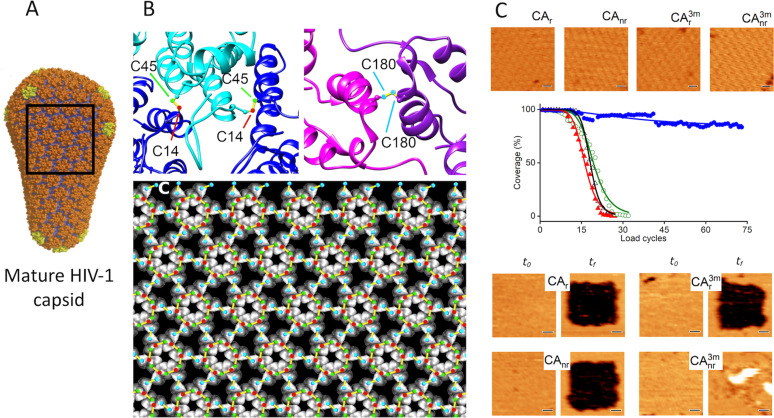
Engineering resistance to fatigue of a virus protein-based nanostructured material. (A) structure of the mature HIV-1 capsid. A square indicates a portion of the hexagonal lattice formed by capsid protein CA subunits. (B) top, protein engineering was used to introduce cysteine residues at specific positions in the CA protein structure. Bottom, under non-reducing conditions, a chainmail of disulfide bonds was formed in the hexagonal 2D lattice self-assembled from engineered CA protein molecules. (C) top, AFM images of protein 2D arrays formed by wt CA under reducing conditions (CA_r_) or non-reducing conditions (CA_nr_), or by engineered CA under reducing conditions (CA^3m^_r_) or non-reducing conditions (CA^3m^_nr_). The scale bars represent 10 nm. Center, remaining % surface covered by the CA protein lattice as a function of the number of indentations applied to induce material fatigue. Red, black and green lines show that the wt CA array, and also the engineered CA array under reducing conditions, were rapidly fatigued, and those arrays were completely disintegrated after <30 indentations under the conditions used. In contrast, the engineered CA array under non-reducing conditions, in which the chainmail of disulfide bonds was formed, showed a dramatically increased resistance to fatigue, as >90% of the array was still formed after ∼70 indentation cycles under the same conditions. Bottom, AFM images of the same protein arrays at the beginning (t_0_) or end (t_f_) of the experiment. Scale bars represent 75 nm. The images show that, after the cyclic indentation process was ended, the indented region of the wt CA array, and of the engineered CA array under reducing conditions, were completely disintegrated (black squares). In contrast, the indented region of the engineered CA array under non-reducing conditions was still nearly fully assembled (no black areas), and had suffered only minor damage. The white areas do not correspond to disrupted patches of the indented protein lattice area; they correspond to some material deposited towards the end of the experiment on the indented CA lattice region. Reproduced from Domínguez-Zotes et al., 2022b with permission from John Wiley & Sons.

The chain mail-like network of covalent bonds that strengthen phage HK97 during its maturation (Section 4.1) inspired the engineering of a 2D nanostructured material based on the hexagonal protein lattice that forms the mature HIV-1 capsid. A complete network of intersubunit disulfide bonds were introduced by rational replacement of some capsid protein residues with cysteines, using protein engineering. The result was a protein-based, nanostructured 2D material with intrinsically improved thermostability, mechanical strength, and resistance to material fatigue, and with no unwanted stiffening (Domínguez-Zotes et al., 2022b) (Fig. 11). That study provided proof of principle for the rational genetic engineering of mechanically strong and fatigue-resistant protein-based nanostructured materials.

## 9 Conclusions

This article has reviewed two decades of AFM-based research on the relationship between virus structure, mechanical properties, and biological function. In the Introduction (Section 1), a number of general questions on the connection between virus biology and mechanical force were formulated. Studies on virus biomechanics have provided some answers to those questions:

i) AFM microscopes allow the controlled application of mechanical force on individual virions, capsids, or other virus components.
ii) application through an AFM tip of a controlled, weak mechanical force on single virus particles, one at a time, allows the visualization of their molecular structure under close to physiological conditions. HS-AFM instruments allow, in addition, the visualization in real time of effector-mediated structural changes of single virus particles.
iii) application of a controlled, high enough mechanical force on single virus particles allows the quantitative determination of their mechanical properties, including stiffness, intrinsic elasticity, strength, brittleness, and resistance to material fatigue.
iv) the mechanical properties of some structurally and functionally very different viruses constitute biologically adaptive traits.
v) many viruses can be subjected to mechanical forces in nature, and some viruses appear to have evolved to withstand, or even use, those natural mechanical forces. In addition, some viruses can exert mechanical forces that contribute to their survival.
vi) quantitative evaluation of the structural and functional responses of virus particles subjected to mechanical forces under controlled conditions in the laboratory is providing novel insights into the relationships between molecular structure and dynamics, physical and physicochemical properties, and biological function of viruses.
vii) mechanical force applied on a virus particle can supply the energy required to trigger changes in virus structure and/or dynamics. Effector-mediated changes in the mechanical properties of a virus particle constitute a signature of changes in particle structure and dynamics that may be biologically relevant. Studies on the mechanical properties of viruses are relevant to understand virus biology, even in cases where natural mechanical forces may not be involved.
viii) application of force on single virus particles allows the investigation of structural and functional differences between individual particles of the same virus species.
ix) the fundamental knowledge acquired on the mechanical properties of viruses is contributing to the development of new biomedical or nanotechnological applications. These include novel antiviral drugs and mechanically strong protein-based or virus-based structured nanomaterials and nanodevices.

## CRediT authorship contribution statement

**Mauricio G. Mateu:** Writing – review & editing, Writing – original draft, Funding acquisition, Conceptualization.

## Declaration of competing interest

The author declares no competing interests.

## Data Availability

This is a review article and all data cited have been published previously
